# Antioxidant and Anti-Inflammatory Effects of Inhaled *Ginkgo biloba* Leaf Essential Oil in a Chronic Stress Mouse Model: Insights from Neuroimmune, Metabolic, and Gut Microbiota Remodeling

**DOI:** 10.3390/plants15182833

**Published:** 2026-09-16

**Authors:** Xingbo Bian, Yu Han, Qi Wang, Jinlong Liu, Zheyuan Gao, Chunyu Zhang, Xiaohang Yang, Xialin Sun

**Affiliations:** School of Pharmaceutical Sciences, Jilin Medical University, Jilin 132013, China; bxb4532@163.com (X.B.); hanyu.jlmu@vip.163.com (Y.H.); wangqicvb@163.com (Q.W.); jinlongliu@jlmu.edu.cn (J.L.); 15934434008@163.com (Z.G.); zcyw3956@sina.com (C.Z.)

**Keywords:** *Ginkgo biloba* leaf essential oil, antioxidant activity, anti-inflammatory activity, chronic stress, neuroinflammation, gut microbiota

## Abstract

Chronic stress induces behavioral abnormalities through coordinated neuroendocrine, inflammatory, oxidative, metabolic, and gut microbial disturbances. Plant-derived volatile fractions may modulate stress-related neurobehavioral responses, yet their antioxidant and anti-inflammatory effects and associated neurobiological mechanisms remain insufficiently characterized. In this study, *Ginkgo biloba* leaf essential oil (GbO) was extracted from air-dried leaves by hydrodistillation, chemically characterized by GC–MS, and evaluated in a chronic unpredictable mild stress (CUMS) mouse model using behavioral, biochemical, molecular, hippocampal metabolomic, and gut microbiota analyses. HS-SPME–GC–MS tentatively identified 70 volatile constituents detected in at least two independent extraction batches, including 65 constituents consistently detected. In CUMS mice, inhaled GbO, particularly at the higher exposure level, attenuated depression- and anxiety-like behavioral abnormalities without altering general locomotor activity. GbO reduced serum corticosterone and peripheral and hippocampal pro-inflammatory cytokines, alleviated hippocampal oxidative imbalance, and decreased Iba-1 and GFAP signals. These changes were accompanied by reduced NF-κB activation, enhanced Nrf2/HO-1-related antioxidant signaling, and improved BDNF/TrkB/CREB-related neurotrophic signaling and PSD95-associated synaptic integrity. Hippocampal metabolomics further showed partial correction of stress-associated disturbances in neurotransmitter, lipid, inflammatory, and energy/redox metabolism, while 16S rRNA sequencing revealed partial restoration of gut microbial diversity and community structure. Collectively, these findings indicate that inhaled GbO exerts antioxidant and anti-inflammatory effects in CUMS mice in association with coordinated neuroimmune, neurotrophic, metabolic, and gut microbial remodeling.

## 1. Introduction

Chronic stress is a major precipitating factor for depression-related neurobehavioral disorders and can induce a broad spectrum of behavioral abnormalities, including anhedonia, behavioral despair, anxiety-like avoidance, and motivational disturbances. Beyond classical monoaminergic dysfunction, chronic stress is increasingly recognized to drive coordinated alterations in neuroendocrine activity, immune signaling, oxidative balance, neuroplasticity, metabolism, and gut microbial ecology [1,2]. In particular, stress-induced peripheral inflammatory activation and hippocampal neuroinflammation can interact with hypothalamic–pituitary–adrenal (HPA) axis activation, glial reactivity, oxidative stress, and impaired synaptic plasticity, thereby forming a multi-level stress–immune–brain regulatory network [3].

The hippocampus is highly vulnerable to chronic stress and plays a central role in mood regulation, stress adaptation, and neuroplasticity. Recent evidence indicates that CUMS can induce depression- and anxiety-like behaviors together with hippocampal microglial activation, inflammatory cytokine upregulation, synaptic loss, and reduced neuronal activity [4]. Astrocytes also participate in depression-related neuroinflammation and neuroplasticity regulation, highlighting the importance of glial responses in stress-associated behavioral dysfunction [5]. These neuroimmune alterations are closely linked to impaired neurotrophic signaling, synaptic structure, and hippocampal metabolic remodeling. Accordingly, integrated analysis of hippocampal glial activation, inflammatory–oxidative signaling, neuroplasticity-related markers, and metabolic changes may provide a more comprehensive understanding of chronic stress-induced behavioral abnormalities [6].

Beyond the central nervous system, the gut microbiota has emerged as an important regulator of stress-related neuroimmune and metabolic responses. Recent evidence indicates that stress-induced microbial changes can promote immune alterations and hippocampal microglial activation, thereby contributing to depression-like behaviors [7]. Remodeling of the gut microbiome has also been shown to promote recovery from stress-induced depression by rescuing hippocampal neurogenesis, while gut microbiome-derived metabolites can reprogram tryptophan metabolism and modulate depression-like behaviors in mice [8,9]. These findings suggest that gut microbial dysbiosis and metabolite remodeling may be closely linked to hippocampal neuroimmune activation and behavioral outcomes. However, the relationships among gut microbial alterations, hippocampal metabolic remodeling, neuroimmune responses, and chronic stress-induced behavioral abnormalities remain incompletely understood.

Volatile plant-derived compounds represent a distinct class of bioactive aromatic exposures that may influence neurobehavioral responses through olfactory pathways, respiratory absorption, systemic circulation, or sensory modulation. Experimental evidence suggests that inhaled aromatic botanical preparations can modulate stress-related behavior and inflammatory responses. Recent work further showed that inhalation aromatherapy ameliorated CUMS-induced depression-like behaviors and olfactory dysfunction, potentially through suppression of inflammatory responses in the peripheral olfactory system [10]. Nevertheless, the neuroimmune and microbiota-associated responses to inhaled medicinal plant-derived volatile fractions remain insufficiently characterized, particularly under chronic stress conditions.

*Ginkgo biloba* L. is a well-known medicinal plant whose leaves contain diverse bioactive constituents with antioxidant, anti-inflammatory, and neuroprotective potential [11]. Recent evidence suggests that *Ginkgo biloba*-based preparations may improve depression-related outcomes and regulate microbiota–metabolite–brain communication, although most available studies focus on standardized non-volatile extracts rather than volatile fractions [12,13]. Compared with these extensively studied non-volatile extracts, the volatile fraction derived from *Ginkgo biloba* leaves remains poorly explored. As a medicinal plant-derived aromatic fraction, *Ginkgo biloba* leaf essential oil (GbO) provides an opportunity to examine whether inhaled botanical volatiles can modulate chronic stress-induced neurobehavioral abnormalities and associated neuroimmune, metabolic, and gut microbial alterations.

In the present study, we used a CUMS mouse model to evaluate the pharmacological effects of inhaled GbO. Chemical profiling, network pharmacology, and molecular docking were used to characterize the volatile mixture and generate candidate molecular hypotheses. The principal experimental focus was the relationship between behavioral outcomes and hippocampal glial activation, inflammatory–oxidative signaling, neurotrophic and synaptic markers, and neurochemical changes, with hippocampal metabolomics, gut microbiota profiling, and correlation-based integration providing systems-level context. This study aimed to determine whether inhaled GbO attenuates chronic stress-induced behavioral abnormalities and to characterize the associated molecular, metabolic, and gut microbial responses.

## 2. Results

### 2.1. Chemical Composition of the Essential Oil

The volatile composition of GbO was characterized by HS-SPME–GC–MS using three independently extracted batches, with Batch 1 corresponding to the batch used in the animal inhalation experiment. Seventy constituents were tentatively identified in at least two batches, of which 65 were consistently detected across all three batches (Table S1). These constituents included terpenes, alcohols, aldehydes, ketones, alkanes, carboxylic acids, esters, and other volatile compounds. Experimental retention indices were compared with literature values reported for HP-5MS or comparable low-polarity 5%-phenyl stationary phases. Sixty-nine assignments showed agreement between the experimental and literature RI values. The assignment of cis-theaspirane showed an appreciable RI discrepancy and was therefore not included in the subsequent computational analysis.

Among the consistently detected constituents with RI agreement, relatively high internal-standard-normalized responses were observed for hexahydrofarnesyl acetone, methyl linolenate, (E)-nerolidol, pentacosane, phytol, and geranylacetone. The identification of 1,8-cineole was supported by match factors of 93.4–94.7% and close agreement between its mean experimental RI and literature RI. Each chromatographic feature was evaluated individually without summing adjacent peaks. The results are presented as tentative identifications and internal-standard-based semiquantitative estimates. Total ion chromatograms from the three independent extraction batches are shown in Figure S1.

### 2.2. Exploratory Network Pharmacology Analysis and Molecular Docking

**Figure 1:**
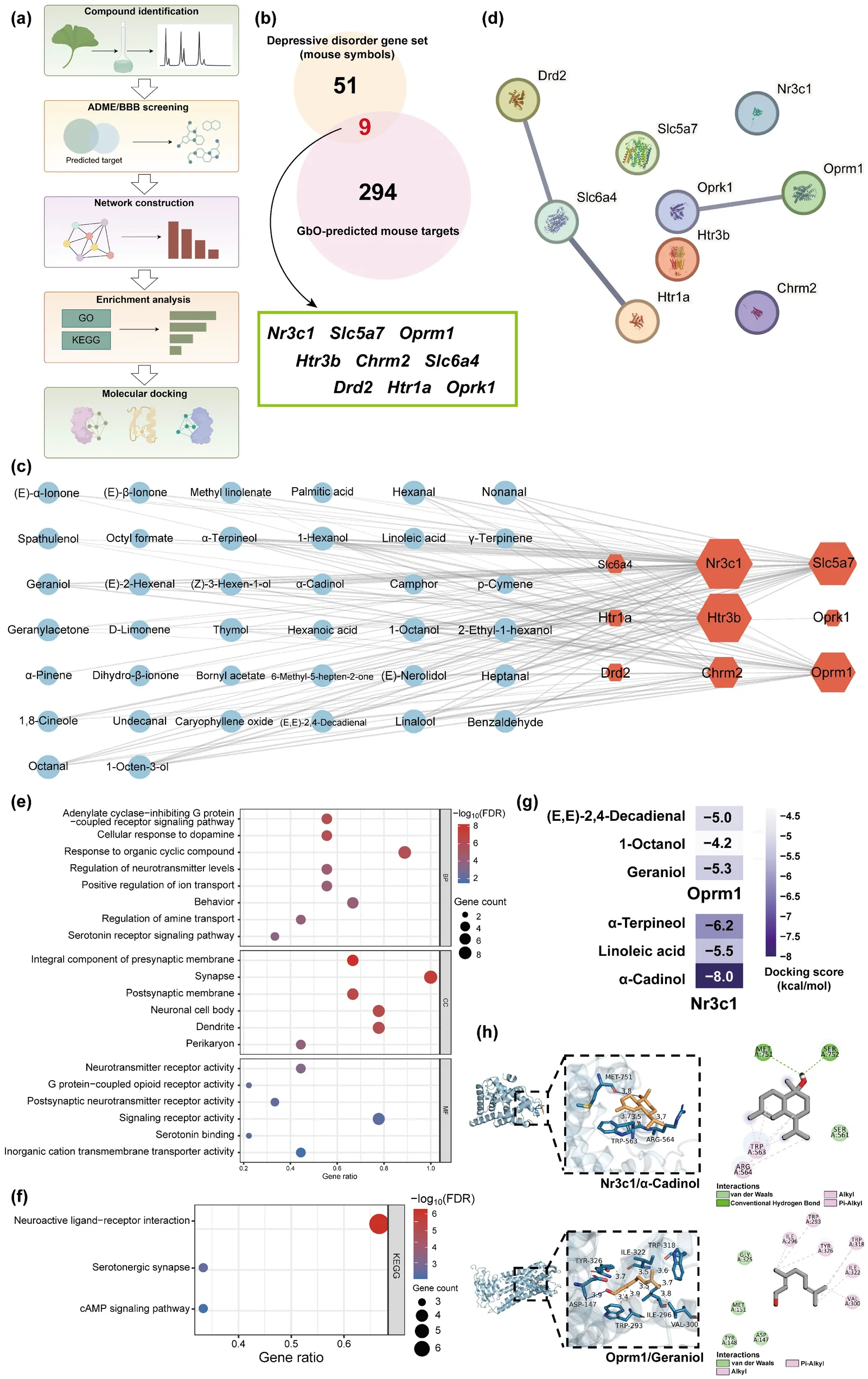
Exploratory network-pharmacology and molecular-docking analyses of *Ginkgo biloba* leaf essential oil. (**a**) Workflow of compound selection, ADME/BBB screening, mouse-target prediction, network construction, functional enrichment, and molecular docking. (**b**) Venn diagram showing the overlap between 303 nonredundant GbO-predicted mouse targets and 60 MGI depressive-disorder-associated genes harmonized to mouse gene symbols. Nine shared genes were identified. (**c**) Compound–target network comprising 38 BBB-permeable compounds, nine shared genes, and 130 predicted compound–target links. Blue circles represent GbO constituents, orange hexagons represent shared genes, and edges indicate predicted associations. Node size is proportional to degree. (**d**) STRING protein–protein interaction network of the nine shared genes at a minimum interaction score of 0.700. Edge thickness represents the STRING interaction score. (**e**) Selected Gene Ontology enrichment terms for biological process (BP), cellular component (CC), and molecular function (MF). (**f**) KEGG pathway enrichment analysis of the shared genes. In (**e**,**f**), dot size represents gene count, dot color represents −log_10_(FDR), and the horizontal axis indicates the gene ratio. (**g**) Molecular-docking scores for three compounds docked with NR3C1 and three compounds docked with OPRM1. Values are expressed in kcal/mol, with more negative values indicating more favorable predicted binding. (**h**) Representative predicted binding poses of α-cadinol with NR3C1 and geraniol with OPRM1. The network-pharmacology and molecular-docking results are presented as exploratory computational predictions and do not establish in vivo exposure or target engagement.

An exploratory network-pharmacology workflow was used to prioritize potential compound–target relationships for subsequent investigation (Figure 1a). Sixty-four tentatively identified GbO constituents that were detected in all three extraction batches and showed agreement between experimental and literature retention indices were evaluated using SwissADME (Table S2). Forty-one constituents were predicted to be BBB permeant and were subsequently submitted to SwissTargetPrediction with *Mus musculus* selected as the target species. After removal of duplicate gene symbols, 303 nonredundant predicted mouse targets were obtained. Comparison with 60 MGI depressive-disorder-associated genes harmonized to mouse gene symbols identified nine shared genes: *Nr3c1*, *Slc5a7*, *Oprm1*, *Htr3b*, *Chrm2*, *Slc6a4*, *Drd2*, *Htr1a*, and *Oprk1* (Figure 1b). The corresponding reduced compound–target network comprised 38 compounds, nine genes, and 130 predicted links (Figure 1c and Table S3). *Nr3c1*, *Slc5a7*, *Oprm1*, *Htr3b*, and *Chrm2* were linked to 34, 27, 24, 23, and 15 compounds, respectively, whereas *Slc6a4*, *Drd2*, *Htr1a*, and *Oprk1* were linked to three, two, one, and one compounds, respectively.

At a STRING interaction-score threshold of 0.700, the protein–protein interaction network contained nine nodes and three edges: *Htr1a*–*Slc6a4* (score = 0.928), *Drd2*–*Slc6a4* (score = 0.785), and *Oprm1*–*Oprk1* (score = 0.762) (Figure 1d). Functional enrichment of the nine shared genes highlighted biological processes related to inhibitory G protein-coupled receptor signaling, responses to dopamine and organic cyclic compounds, neurotransmitter regulation, and serotonin receptor signaling. Enriched cellular-component terms included the presynaptic membrane, synapse, postsynaptic membrane, neuronal cell body, dendrite, and perikaryon, while molecular-function terms included neurotransmitter-receptor and signaling-receptor activities (Figure 1e). KEGG analysis identified neuroactive ligand–receptor interaction, serotonergic synapse, and the cAMP signaling pathway (Figure 1f).

Molecular docking was then used to examine selected predicted ligand–target pairs involving NR3C1 and OPRM1. The docking scores for α-cadinol, α-terpineol, and linoleic acid with NR3C1 were −8.0, −6.2, and −5.5 kcal/mol, respectively. The corresponding scores for geraniol, (E,E)-2,4-decadienal, and 1-octanol with OPRM1 were −5.3, −5.0, and −4.2 kcal/mol, respectively (Figure 1g). Representative binding poses are shown for α-cadinol–NR3C1 and geraniol–OPRM1 (Figure 1h). Collectively, these analyses prioritize candidate compound–target relationships and signaling pathways as testable hypotheses rather than evidence of in vivo exposure or target engagement.

### 2.3. Antidepressant-like Effects of Ginkgo biloba Leaf Essential Oil in CUMS Mice

**Figure 2:**
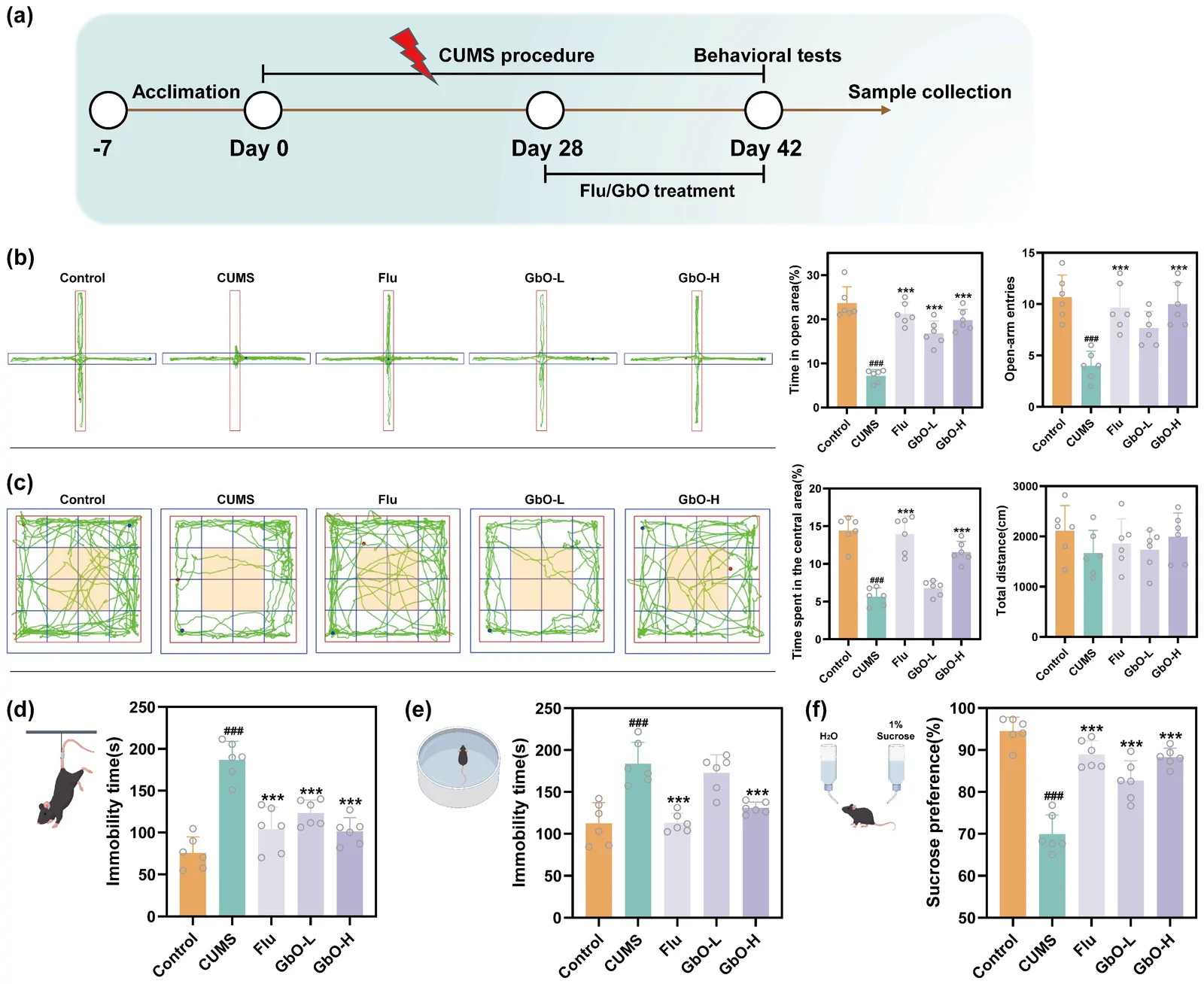
*Ginkgo biloba* leaf essential oil alleviated depression- and anxiety-like behaviors in CUMS mice. (**a**) Experimental timeline showing CUMS exposure, fluoxetine/GbO treatment, behavioral testing, and sample collection. (**b**) Representative movement trajectories and quantitative analysis of the elevated plus maze (EPM), including time spent in the open arms and open-arm entries. (**c**) Representative movement trajectories and quantitative analysis of the open field test (OFT), including time spent in the central area and total distance traveled. (**d**) Immobility time in the tail suspension test (TST). (**e**) Immobility time in the forced swimming test (FST). (**f**) Sucrose preference in the sucrose preference test (SPT). Data are presented as mean ± SD (n = 6 mice per group). Statistical significance was determined by one-way ANOVA followed by Tukey’s test. ### *p* < 0.001 vs. Control group; *** *p* < 0.001 vs. CUMS group.

To evaluate the neurobehavioral effects of GbO in vivo, mice were subjected to CUMS for 42 days, and Flu or GbO treatment was administered from day 28 to day 42 (Figure 2a). A battery of behavioral tests was then performed to assess anxiety-like behavior, exploratory activity, behavioral despair, and anhedonia.

In the EPM test (Figure 2b), CUMS exposure significantly reduced both the time spent in the open arms and the number of open-arm entries compared with the Control group, indicating enhanced anxiety-like behavior. Flu treatment markedly reversed these changes. GbO treatment also increased open-arm exploration, with the GbO-H group showing a more pronounced effect than the GbO-L group.

In the OFT (Figure 2c), CUMS mice spent significantly less time in the central area than Control mice, further supporting the occurrence of anxiety-like behavior under chronic stress. Flu and GbO-H treatment significantly increased the time spent in the central area, whereas GbO-L showed a weaker effect. No significant difference in total distance traveled was observed among the groups, suggesting that the behavioral changes were not attributable to altered general locomotor activity.

In the TST (Figure 2d), CUMS significantly increased immobility time, indicating enhanced behavioral despair. Flu and both GbO exposure levels significantly reduced immobility time, with GbO-H showing a clear antidepressant-like effect. Similarly, in the FST (Figure 2e), CUMS prolonged immobility time, whereas Flu and GbO-H markedly reduced immobility time; the effect of GbO-L was less pronounced. In the SPT (Figure 2f), CUMS significantly decreased sucrose preference, indicating anhedonia-like behavior. Flu and GbO treatment restored sucrose preference, with GbO-H showing a stronger effect than GbO-L.

Together, these behavioral results indicate that CUMS induced robust depression- and anxiety-like behavioral abnormalities in mice, while GbO treatment, particularly at the higher exposure level, partially alleviated these chronic stress-induced behavioral deficits.

### 2.4. Ginkgo biloba Leaf Essential Oil Alleviated Hippocampal Morphological Alterations and Biochemical Abnormalities in CUMS Mice

To further explore the neurobiological changes associated with the behavioral effects of GbO, hippocampal morphology, dendritic spine density, inflammatory responses, oxidative stress, and neurochemical indicators were examined in CUMS mice.

**Figure 3:**
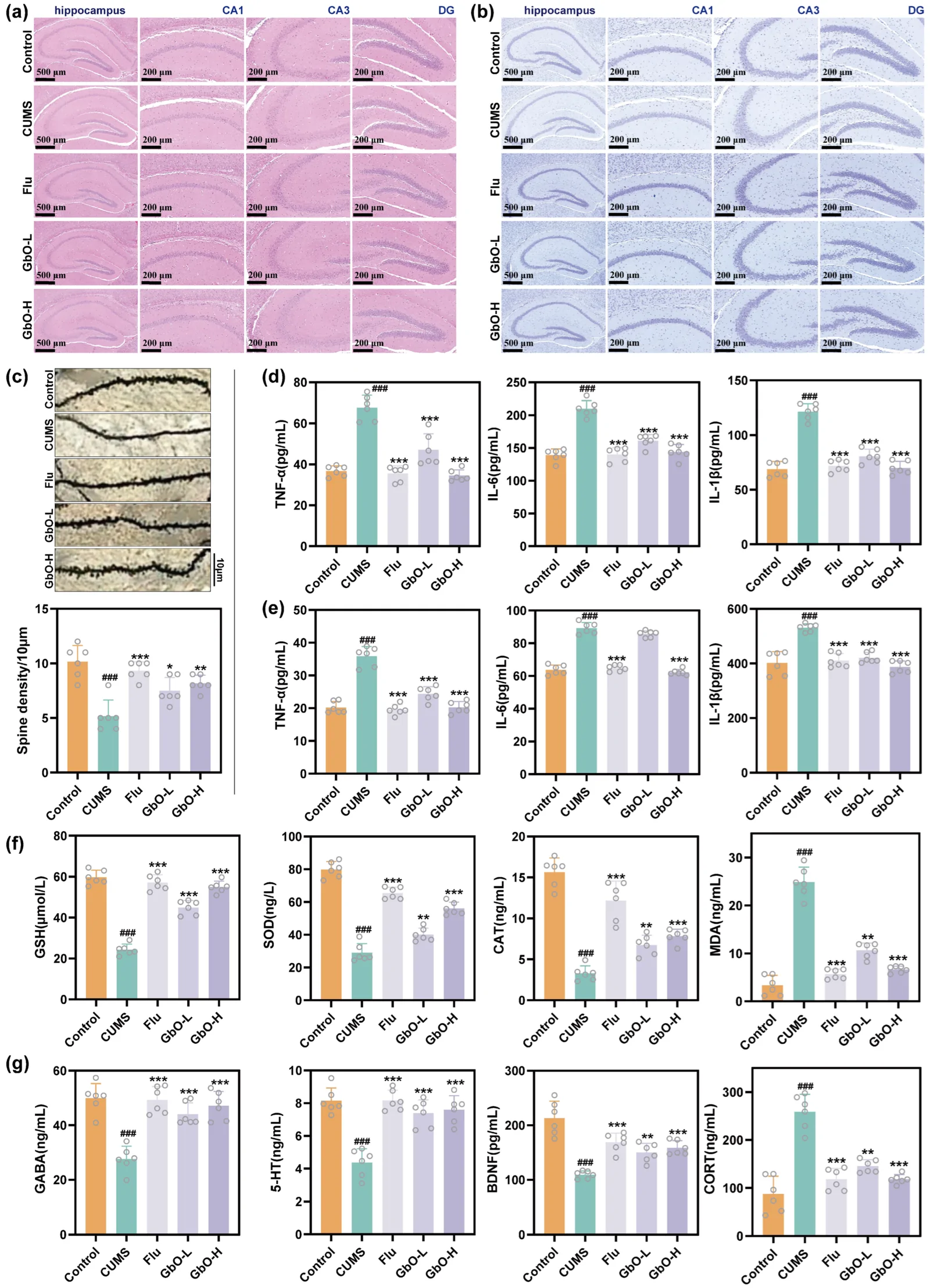
*Ginkgo biloba* leaf essential oil alleviated hippocampal morphological alterations, dendritic spine loss, inflammatory responses, oxidative imbalance, and neurochemical disturbances in CUMS mice. (**a**) Representative H&E staining images of the hippocampus and the CA1, CA3, and DG subregions. (**b**) Representative Nissl staining images of the hippocampus and the CA1, CA3, and DG subregions. (**c**) Representative Golgi staining images and quantification of dendritic spine density in hippocampal neurons. (**d**) Serum levels of TNF-α, IL-6, and IL-1β. (**e**) Levels of TNF-α, IL-6, and IL-1β in hippocampal homogenates. (**f**) Hippocampal oxidative stress-related indices, including GSH, SOD, CAT, and MDA. (**g**) Levels of GABA, 5-HT, and BDNF in hippocampal homogenates and serum CORT levels. Data are presented as mean ± SD (n = 6 mice per group). Statistical significance was determined by one-way ANOVA followed by Tukey’s test. ### *p* < 0.001 vs. Control group; * *p* < 0.05, ** *p* < 0.01, and *** *p* < 0.001 vs. CUMS group.

H&E staining showed that neurons in the hippocampal CA1, CA3, and DG regions of Control mice were relatively well organized with clear morphology. Compared with the Control group, CUMS mice showed observable hippocampal morphological alterations, including less organized neuronal arrangement in these subregions (Figure 3a). Flu and GbO treatment partially improved these stress-associated morphological changes, with a more evident effect observed in the GbO-H group. Similarly, Nissl staining showed attenuated Nissl staining intensity and less compact neuronal organization in the hippocampus of CUMS mice, whereas Flu and GbO treatment partially restored Nissl staining patterns (Figure 3b). Golgi staining further showed that CUMS significantly decreased hippocampal dendritic spine density, indicating impaired synaptic structural plasticity. Flu and GbO treatment significantly increased dendritic spine density compared with the CUMS group (Figure 3c).

As shown in Figure 3d, serum levels of TNF-α, IL-6, and IL-1β were significantly elevated in CUMS mice, whereas Flu and GbO treatment reduced these pro-inflammatory cytokines. Consistently, TNF-α, IL-6, and IL-1β levels in hippocampal homogenates were also increased after CUMS exposure and were decreased by Flu and GbO treatment (Figure 3e). CUMS also induced hippocampal oxidative imbalance, as reflected by decreased GSH, SOD, and CAT levels and increased MDA levels. These alterations were reversed by Flu and GbO treatment, with GbO-H generally showing a stronger effect (Figure 3f). In addition, CUMS caused neurochemical disturbances, including decreased hippocampal GABA, 5-HT, and BDNF levels, together with increased serum CORT levels. Flu and GbO treatment partially corrected these changes, especially in the GbO-H group (Figure 3g).

Overall, these findings indicate that GbO alleviated CUMS-associated hippocampal morphological alterations, dendritic spine loss, peripheral and hippocampal inflammatory responses, oxidative imbalance, and neurochemical disturbances.

### 2.5. Ginkgo biloba Leaf Essential Oil Attenuated Hippocampal Glial Activation and Inflammatory–Oxidative Signaling Alterations in CUMS Mice

To further investigate whether the behavioral effects of GbO were associated with hippocampal neuroinflammatory regulation, immunofluorescence staining, qPCR, and Western blot analyses were performed to evaluate glial activation, inflammatory gene expression, and inflammation- and antioxidant-related signaling proteins in the hippocampus.

**Figure 4:**
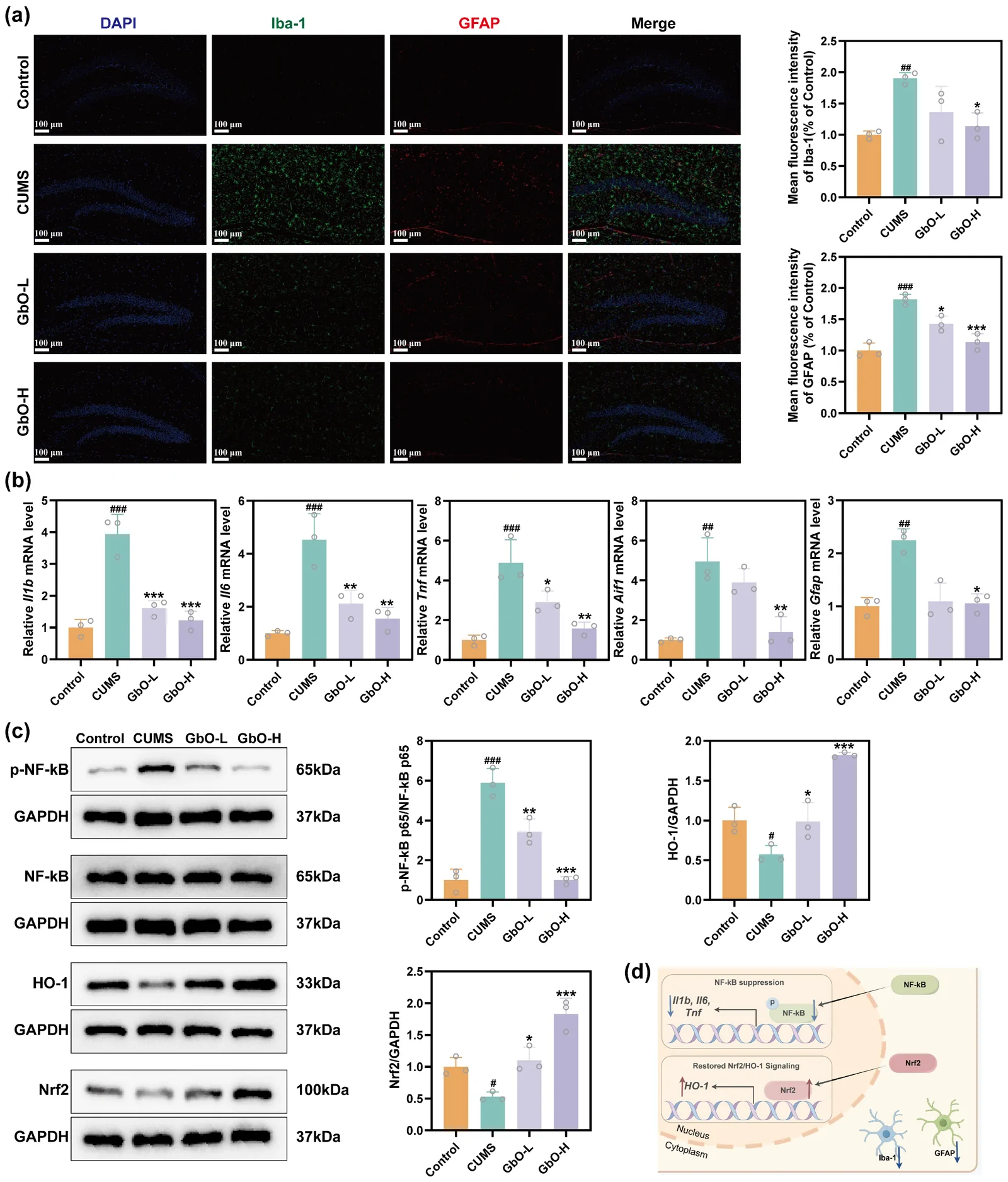
*Ginkgo biloba* leaf essential oil attenuated hippocampal glial activation and inflammatory–oxidative signaling alterations in CUMS mice. (**a**) Representative immunofluorescence images of DAPI, Iba-1, GFAP, and merged signals in the hippocampus, together with quantitative analysis of Iba-1 and GFAP fluorescence intensity. (**b**) Relative mRNA expression levels of *Il1b*, *Il6*, *Tnf*, *Aif1*, and *Gfap* in the hippocampus. (**c**) Representative Western blot bands and quantitative analysis of p-NF-κB p65/NF-κB p65, HO-1, and Nrf2 protein levels in the hippocampus. (**d**) Schematic summary of the proposed anti-neuroinflammatory effects of GbO in the hippocampus. Black arrows indicate the proposed regulatory relationships, whereas upward and downward arrows indicate increases and decreases, respectively. Data are presented as mean ± SD (n = 3 mice per group). Statistical significance was determined by one-way ANOVA followed by Tukey’s test. # *p* < 0.05, ## *p* < 0.01, and ### *p* < 0.001 vs. Control group; * *p* < 0.05, ** *p* < 0.01, and *** *p* < 0.001 vs. CUMS group.

Immunofluorescence staining showed that the fluorescence intensities of Iba-1 and GFAP were markedly increased in the hippocampus of CUMS mice compared with the Control group, indicating enhanced microglial activation and astrocyte reactivity under chronic stress (Figure 4a). GbO treatment reduced both Iba-1 and GFAP fluorescence intensities, with the GbO-H group showing a more pronounced effect.

Consistent with the immunofluorescence results, qPCR analysis showed that CUMS significantly upregulated the mRNA expression levels of *Il1b*, *Il6*, *Tnf*, *Aif1*, and *Gfap* in the hippocampus (Figure 4b). GbO treatment decreased the expression of these inflammatory and glial activation-related genes, particularly in the GbO-H group.

Western blot analysis further showed that CUMS increased the p-NF-κB/NF-κB p65 ratio, while decreasing the protein levels of HO-1 and Nrf2 in the hippocampus (Figure 4c). GbO treatment reduced NF-κB phosphorylation and increased Nrf2 and HO-1 protein expression, suggesting that GbO may help rebalance hippocampal inflammatory and antioxidant responses under chronic stress.

Together, these findings indicate that GbO attenuated CUMS-induced hippocampal glial activation and inflammatory–oxidative signaling alterations, which may contribute to its neurobehavioral effects (Figure 4d).

### 2.6. Ginkgo biloba Leaf Essential Oil Was Associated with Improved Hippocampal Neuronal Status and Synaptic Plasticity in CUMS Mice

To further determine whether GbO-associated behavioral improvement was accompanied by changes in hippocampal neuronal status and synaptic plasticity, immunofluorescence staining, qPCR, and Western blot analyses were performed to evaluate neurotrophic and synaptic plasticity-related markers in the hippocampus.

**Figure 5:**
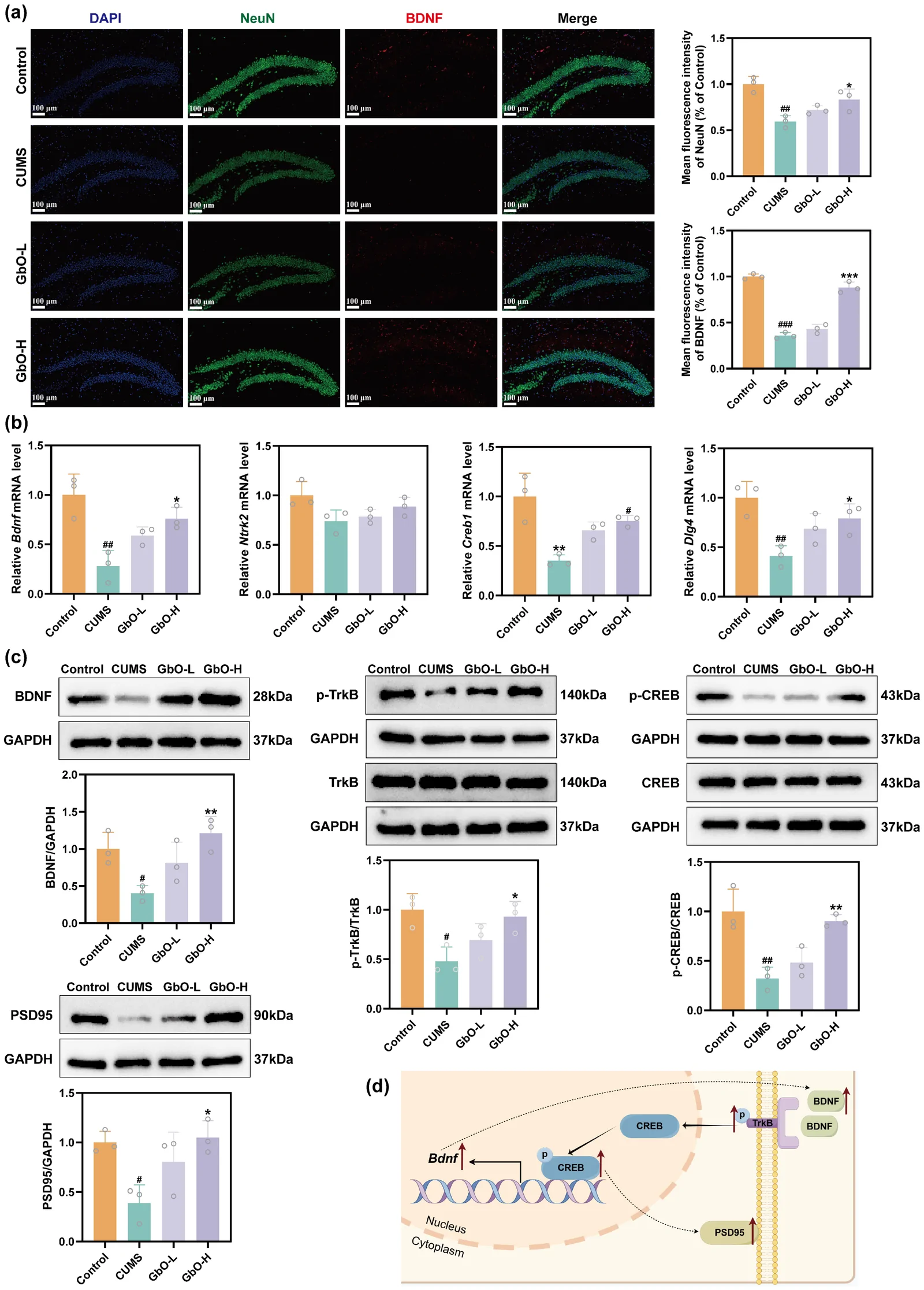
*Ginkgo biloba* leaf essential oil was associated with improved hippocampal neurotrophic signaling and synaptic plasticity in CUMS mice. (**a**) Representative immunofluorescence images of DAPI, NeuN, BDNF, and merged signals in the hippocampus, together with quantitative analysis of NeuN and BDNF fluorescence intensities. (**b**) Relative mRNA expression levels of *Bdnf*, *Ntrk2*, *Creb1*, and *Dlg4* in the hippocampus. (**c**) Representative Western blot bands and quantitative analysis of BDNF, PSD95, p-TrkB/TrkB, and p-CREB/CREB protein levels in the hippocampus. (**d**) Schematic summary of the proposed mechanism by which GbO improves hippocampal neurotrophic signaling and synaptic plasticity through restoration of the BDNF/TrkB/CREB axis and enhancement of PSD95-associated synaptic remodeling. Solid and dashed black arrows indicate the proposed regulatory relationships, whereas upward arrows indicate increases. Data are presented as mean ± SD (n = 3 mice per group). Statistical significance was determined by one-way ANOVA followed by Tukey’s test. # *p* < 0.05, ## *p* < 0.01, and ### *p* < 0.001 vs. Control group; * *p* < 0.05, ** *p* < 0.01, and *** *p* < 0.001 vs. CUMS group.

Immunofluorescence staining showed that the fluorescence intensities of NeuN and BDNF were markedly decreased in the hippocampus of CUMS mice compared with the Control group, indicating impaired neuronal status and reduced neurotrophic support under chronic stress (Figure 5a). GbO treatment increased the fluorescence signals of both NeuN and BDNF, with the GbO-H group showing a more pronounced effect.

Consistent with the immunofluorescence results, qPCR analysis showed that CUMS significantly downregulated the mRNA expression levels of *Bdnf*, *Creb1*, and *Dlg4* in the hippocampus, whereas *Ntrk2* showed a decreasing trend (Figure 5b). GbO-H significantly restored *Bdnf*, *Creb1*, and *Dlg4* expression and tended to increase *Ntrk2* expression, suggesting partial recovery of neurotrophic and synaptic plasticity-related transcriptional changes.

Western blot analysis further showed that CUMS decreased BDNF and PSD95 protein levels, as well as the phosphorylation ratios of TrkB and CREB, compared with the Control group (Figure 5c). GbO treatment, particularly GbO-H, restored BDNF and PSD95 expression and increased the p-TrkB/TrkB and p-CREB/CREB ratios. These findings suggest that GbO-associated improvement in hippocampal neuronal status and synaptic plasticity may involve BDNF/TrkB/CREB-related neurotrophic signaling and PSD95-associated synaptic integrity.

Together, these results indicate that GbO partially alleviated CUMS-induced impairment of hippocampal neuronal status and synaptic plasticity, which may contribute to its neurobehavioral effects (Figure 5d).

### 2.7. Ginkgo biloba Leaf Essential Oil Modulated Global Hippocampal Metabolic Profiles in CUMS Mice

To further elucidate the metabolic alterations associated with the neurobehavioral effects of GbO, untargeted metabolomic analysis of hippocampal tissues was performed in the Control, CUMS, and GbO-H groups.

**Figure 6:**
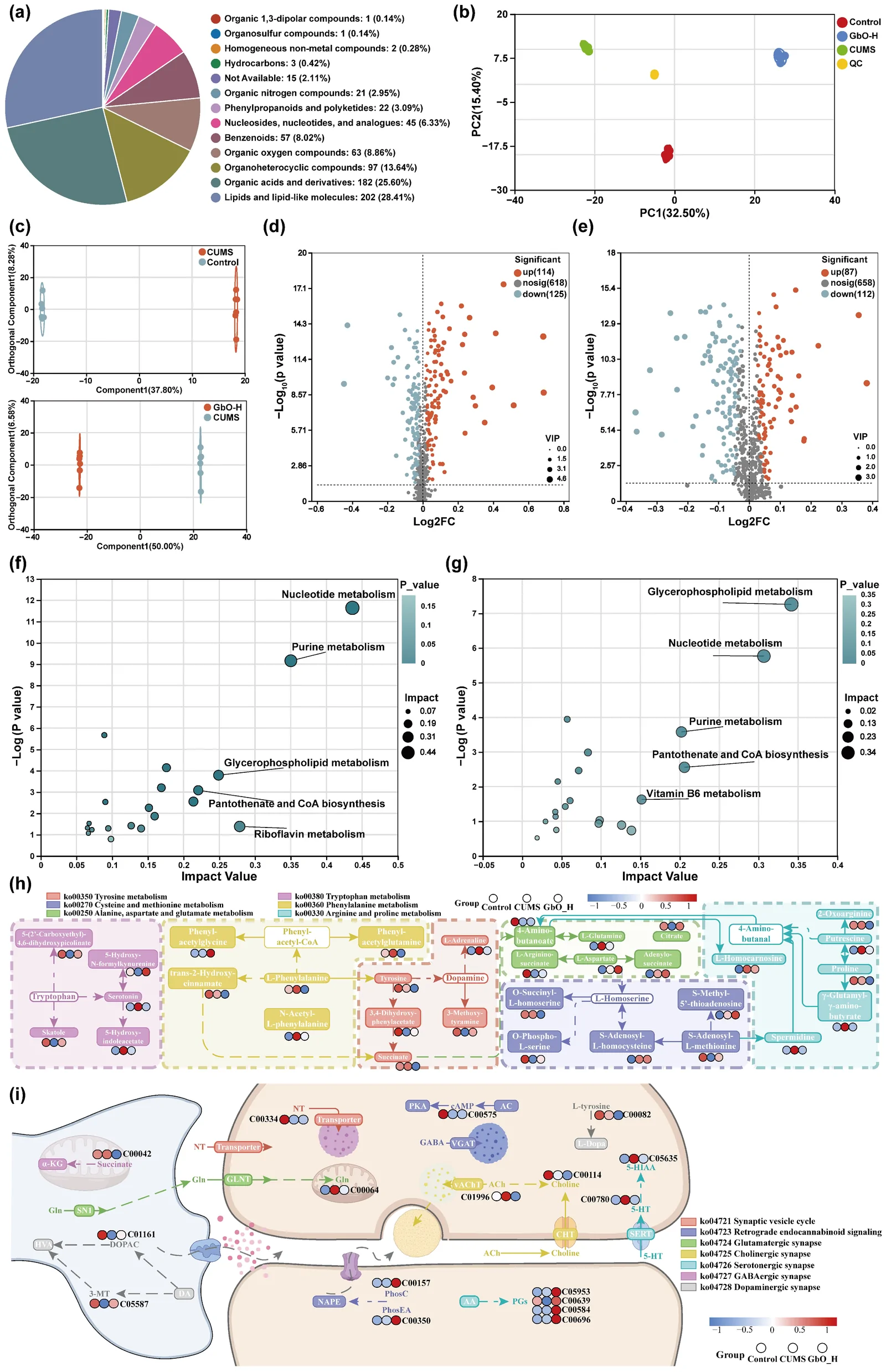
*Ginkgo biloba* leaf essential oil modulated global hippocampal metabolomic profiles in CUMS mice. (**a**) Classification of identified metabolites based on chemical categories. (**b**) PCA score plot showing the distribution of the Control, CUMS, and GbO-H groups, together with QC samples. (**c**) OPLS-DA score plots illustrating metabolic separation between the Control and CUMS groups and between the CUMS and GbO-H groups. (**d**,**e**) Volcano plots showing significantly altered metabolites in the indicated comparisons. (**f**,**g**) KEGG pathway enrichment analysis of differential metabolites. (**h**) Global metabolic network summary highlighting the major metabolic modules altered by CUMS and modulated by GbO-H. (**i**) Summary of neurotransmission- and synaptic function-related metabolic alterations in the hippocampus.

The overall distribution of identified metabolites showed that lipids and lipid-like molecules, organic acids and derivatives, and organoheterocyclic compounds constituted the major metabolite classes in the hippocampus (Figure 6a). Multivariate analysis revealed distinct metabolic profiles among the Control, CUMS, and GbO-H groups. PCA score plots showed clear clustering patterns, with QC samples tightly clustered, indicating acceptable analytical stability (Figure 6b). OPLS-DA further showed metabolic separation between the Control and CUMS groups, as well as between the CUMS and GbO-H groups (Figure 6c), suggesting that CUMS induced broad hippocampal metabolic disturbances that were partially modulated by GbO-H treatment.

Differential metabolite analysis identified numerous significantly altered metabolites in the CUMS vs. Control and GbO-H vs. CUMS comparisons (Figure 6d,e). Compared with the Control group, CUMS mice exhibited substantial hippocampal metabolic alterations, whereas GbO-H treatment oppositely regulated a subset of these altered metabolic features. KEGG pathway enrichment analysis showed that the differential metabolites were mainly involved in nucleotide metabolism, purine metabolism, glycerophospholipid metabolism, pantothenate and CoA biosynthesis, and vitamin B6 metabolism (Figure 6f,g). In addition, global metabolic network analysis suggested that CUMS-associated hippocampal metabolic disturbances were related to amino acid metabolism, neurotransmitter-related pathways, and synaptic function, including tryptophan metabolism, glutamate metabolism, serotonergic synapse, GABAergic synapse, and dopaminergic synapse (Figure 6h,i).

Together, these results indicate that GbO-H partially corrected CUMS-associated hippocampal metabolic disturbances, particularly in pathways related to neurotransmitter regulation, lipid metabolism, and cellular energy homeostasis.

### 2.8. Ginkgo biloba Leaf Essential Oil Regulated Key Hippocampal Metabolic Alterations Induced by CUMS

To further identify key metabolic features modulated by GbO-H, we focused on differential metabolites that showed opposite changing patterns in the CUMS vs. Control and GbO-H vs. CUMS comparisons.

**Figure 7:**
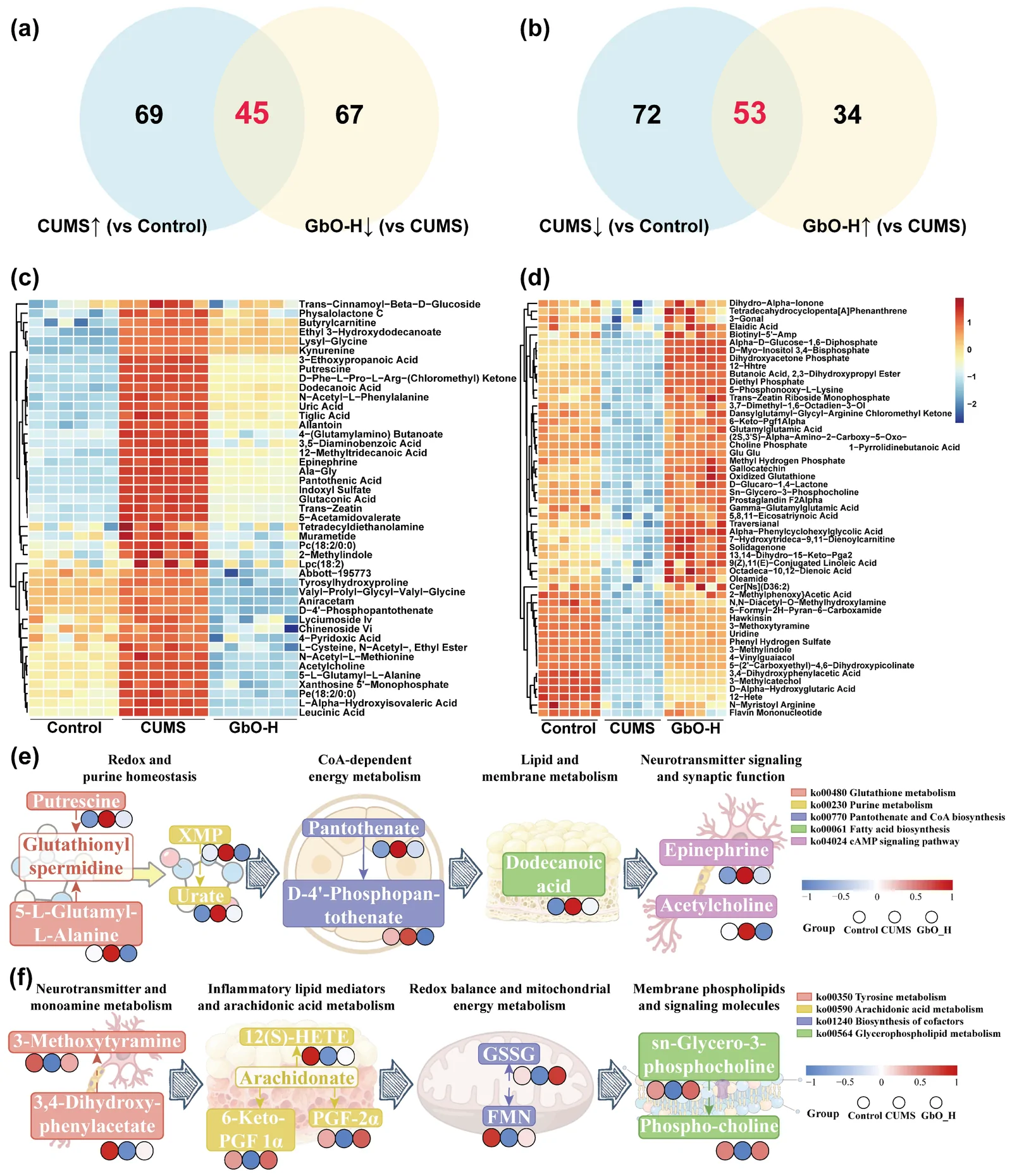
*Ginkgo biloba* leaf essential oil oppositely regulated key hippocampal metabolites altered by CUMS. (**a**) Venn diagram showing metabolites that were increased in the CUMS group relative to the Control group and decreased in the GbO-H group relative to the CUMS group. (**b**) Venn diagram showing metabolites that were decreased in the CUMS group relative to the Control group and increased in the GbO-H group relative to the CUMS group. Upward and downward arrows indicate increased and decreased metabolite levels, respectively. (**c**,**d**) Heatmaps showing the relative abundance patterns of metabolites reversed by GbO-H among the Control, CUMS, and GbO-H groups. (**e**) Functional integration of representative metabolites that were increased in the CUMS group and decreased after GbO-H treatment, mainly associated with redox and purine homeostasis, CoA-dependent energy metabolism, lipid and membrane metabolism, and neurotransmitter signaling and synaptic function. (**f**) Functional integration of representative metabolites that were decreased in the CUMS group and restored after GbO-H treatment, mainly associated with neurotransmitter and monoamine metabolism, inflammatory lipid mediators and arachidonic acid metabolism, redox balance and mitochondrial energy metabolism, and membrane phospholipids and signaling molecules. Thin arrows indicate the depicted metabolic relationships, whereas large block arrows indicate connections between functional modules.

Venn analysis showed that 45 metabolites were significantly upregulated in the CUMS group compared with the Control group and subsequently downregulated after GbO-H treatment (Figure 7a). In contrast, 53 metabolites were significantly downregulated in the CUMS group and increased after GbO-H treatment (Figure 7b). These results indicate that GbO-H oppositely regulated a subset of CUMS-altered hippocampal metabolites.

Heatmap analysis further showed that the abundance patterns of these oppositely regulated metabolites in the GbO-H group partially shifted toward those of the Control group, whereas the CUMS group showed a distinct metabolic profile (Figure 7c,d). This finding suggests that GbO-H partially corrected chronic stress-associated hippocampal metabolic abnormalities.

Functional integration analysis showed that metabolites increased by CUMS but decreased by GbO-H were mainly associated with redox and purine homeostasis, CoA-dependent energy metabolism, lipid and membrane metabolism, and neurotransmitter signaling and synaptic function (Figure 7e). In contrast, metabolites decreased by CUMS but increased by GbO-H were mainly involved in neurotransmitter and monoamine metabolism, inflammatory lipid mediators and arachidonic acid metabolism, redox balance and mitochondrial energy metabolism, as well as membrane phospholipids and signaling molecules (Figure 7f).

Together, these results indicate that GbO-H partially corrected CUMS-associated hippocampal metabolic disturbances by oppositely regulating key metabolites related to neurotransmitter metabolism, inflammatory lipid signaling, membrane remodeling, and energy/redox homeostasis.

### 2.9. Ginkgo biloba Leaf Essential Oil Reshaped Gut Microbiota Composition and Microbial Correlation Networks in CUMS Mice

To investigate whether gut microbiota alterations were associated with the neurobehavioral effects of GbO, 16S rRNA gene sequencing was performed on fecal samples from the Control, CUMS, and GbO-H groups.

**Figure 8:**
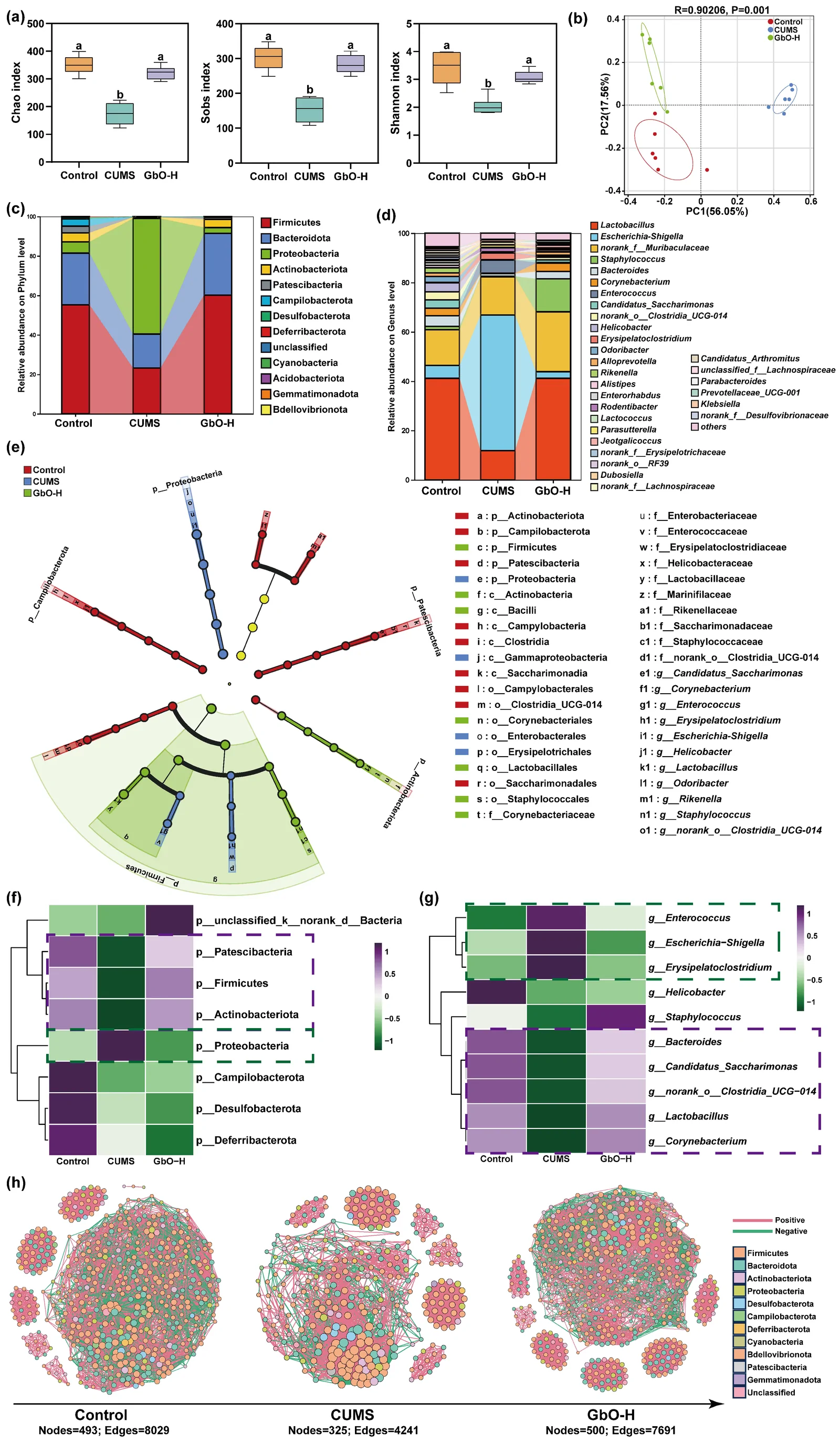
*Ginkgo biloba* leaf essential oil reshaped gut microbiota composition and microbial correlation networks in CUMS mice. (**a**) Alpha diversity indices, including Chao, Sobs, and Shannon indices, of gut microbiota among the Control, CUMS, and GbO-H groups. Differences among groups were evaluated by one-way ANOVA followed by Tukey’s multiple-comparison test. Different lowercase letters indicate significant differences among groups (*p* < 0.05). (**b**) Principal coordinate analysis (PCoA) score plot showing differences in microbial community structure among groups based on Bray–Curtis distances. Group separation was evaluated by ANOSIM. (**c**) Relative abundance of gut microbiota at the phylum level. (**d**) Relative abundance of gut microbiota at the genus level. (**e**) LEfSe analysis identifying differentially abundant taxa among groups. (**f**,**g**) Heatmaps showing differentially abundant taxa at the phylum and genus levels. (**h**) Microbial co-occurrence networks showing differences in community interaction patterns among groups. Nodes represent microbial taxa, node colors indicate phylum-level classification, and edges represent significant Spearman correlations. Red and green edges indicate positive and negative correlations, respectively.

Alpha diversity analysis showed that the Chao, Sobs, and Shannon indices were significantly decreased in the CUMS group compared with the Control group, indicating reduced microbial richness and diversity. GbO-H treatment significantly increased these indices compared with the CUMS group, suggesting partial recovery of gut microbial alpha diversity (Figure 8a). PCoA based on Bray–Curtis distances revealed clear separation among the Control, CUMS, and GbO-H groups, and ANOSIM confirmed significant differences in microbial community structure (R = 0.90206, *p* = 0.001) (Figure 8b).

At the phylum level, CUMS markedly altered gut microbial composition, characterized by an increased relative abundance of Proteobacteria and decreased relative abundances of Firmicutes, Actinobacteriota, and Patescibacteria. GbO-H treatment partially corrected these stress-associated phylum-level changes (Figure 8c,f). At the genus level, CUMS increased the relative abundances of Enterococcus, Escherichia-Shigella, and Erysipelatoclostridium, while reducing several genera, including Lactobacillus, Bacteroides, Candidatus_Saccharimonas, norank_o__Clostridia_UCG-014, and Corynebacterium. GbO-H treatment partially shifted these taxa toward the Control pattern, as shown by the genus-level composition and heatmap analyses (Figure 8d,g).

LEfSe analysis further identified differentially enriched taxa among the Control, CUMS, and GbO-H groups, supporting distinct microbial signatures under chronic stress and GbO-H treatment (Figure 8e). In particular, CUMS-associated enrichment of Proteobacteria-related taxa and stress-associated genera was accompanied by reductions in several taxa that were partially restored in the GbO-H group. These results suggest that GbO-H selectively reshaped specific microbial taxa altered by CUMS rather than inducing a nonspecific shift in the gut microbial community.

Microbial correlation network analysis further showed that CUMS reduced network size and connectivity, as reflected by decreases in nodes, edges, and average degree, together with an increase in network diameter (Figure 8h and Table S4). The Control network contained 493 nodes and 8029 edges, whereas the CUMS network contained 325 nodes and 4241 edges. After GbO-H treatment, the network contained 500 nodes and 7691 edges, suggesting partial restoration of microbial correlation complexity. Because these networks were constructed based on Spearman correlations, they should be interpreted as microbial association patterns rather than direct ecological interactions.

Together, these results indicate that GbO-H partially corrected CUMS-associated gut microbiota dysbiosis by reshaping microbial diversity, community composition, and microbial correlation network structure.

### 2.10. Correlation-Based Multi-Omics Analysis Revealed Associations Among Gut Microbiota, Hippocampal Metabolites, and Neurobiological Indicators

To further explore the relationships among gut microbiota, hippocampal metabolites, and host neurobiological indicators, correlation-based multi-omics analyses were performed.

**Figure 9:**
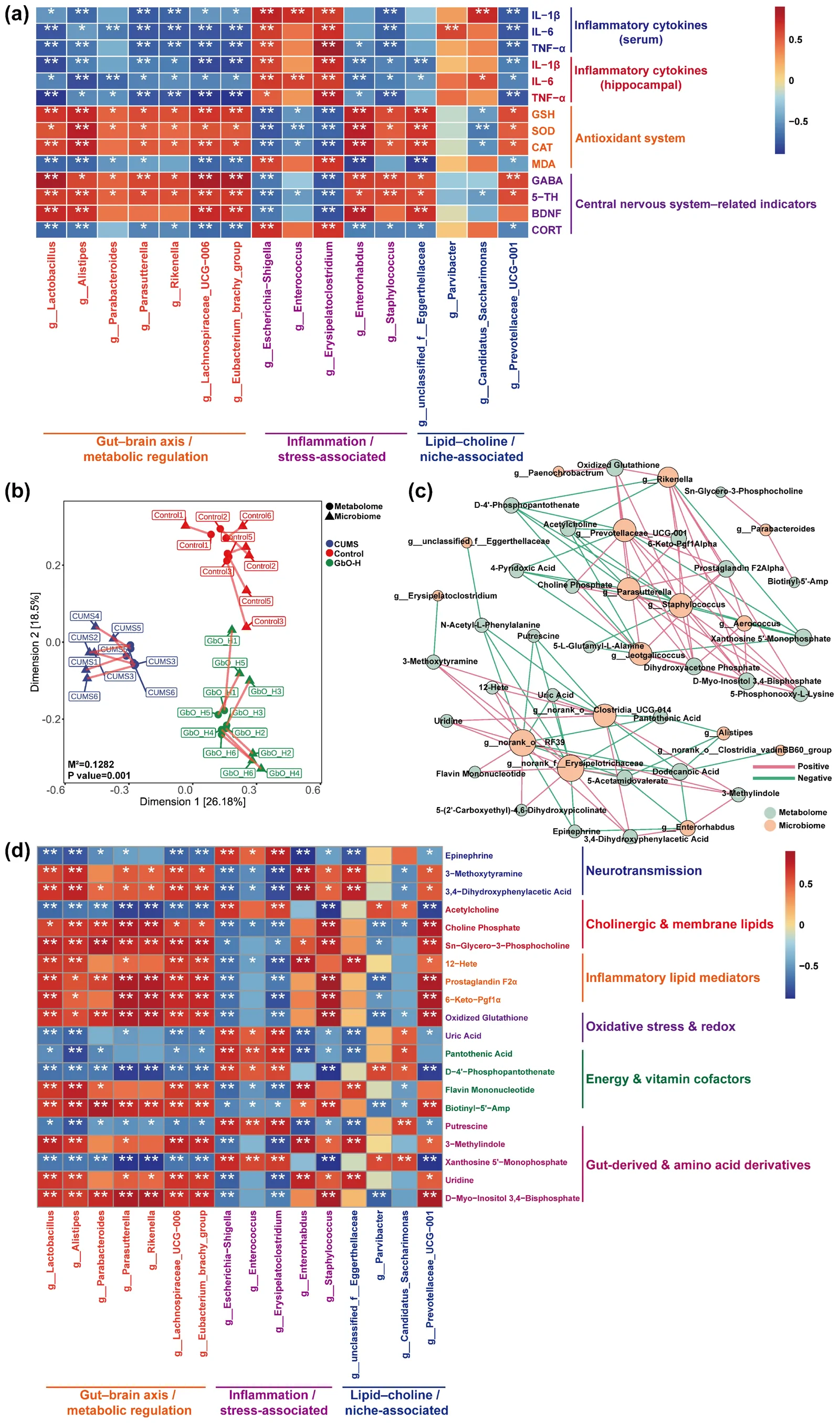
Multi-omics integration analysis linking gut microbiota, hippocampal metabolites, and neurobiological indicators in CUMS mice. (**a**) Correlation heatmap showing associations between gut microbial genera and inflammatory cytokines, antioxidant indices, and neurobiological indicators. Correlations were calculated using Spearman’s rank correlation analysis. Color intensity indicates the correlation coefficient. * *p* < 0.05 and ** *p* < 0.01. (**b**) Procrustes analysis showing the concordance between gut microbiota and hippocampal metabolomic profiles. (**c**) Correlation network integrating gut microbial genera and representative hippocampal metabolites. Red and green lines indicate positive and negative correlations, respectively. (**d**) Correlation heatmap showing associations between gut microbial genera and representative metabolites involved in neurotransmission, cholinergic signaling and membrane lipids, inflammatory lipid mediators, oxidative stress and redox balance, energy and vitamin cofactors, and gut-derived or amino acid-related metabolites. Correlations were calculated using Spearman’s rank correlation analysis. Color intensity indicates the correlation coefficient. * *p* < 0.05 and ** *p* < 0.01.

Spearman correlation heatmap analysis showed that multiple gut microbial genera were significantly associated with inflammatory cytokines, oxidative stress-related indices, and neurobiological indicators (Figure 9a). For example, Lactobacillus showed negative correlations with pro-inflammatory cytokines, including IL-1β, IL-6, and TNF-α, and positive correlations with neurobiological factors such as GABA, 5-HT, and BDNF. In contrast, several CUMS-enriched genera displayed opposite correlation patterns. These results suggest that stress-associated microbial shifts were closely related to peripheral and hippocampal biochemical changes.

Procrustes analysis further showed significant concordance between gut microbiota profiles and hippocampal metabolomic profiles (M^2^ = 1.1774, *p* = 0.001), indicating that microbial community variation was significantly associated with hippocampal metabolic remodeling (Figure 9b).

Correlation network analysis integrating gut microbial genera and representative hippocampal metabolites revealed extensive positive and negative associations (Figure 9c). Several metabolites related to neurotransmitter metabolism, lipid signaling, and oxidative stress were associated with specific microbial taxa, suggesting potential microbiota–metabolite links under CUMS and GbO-H treatment conditions. Further correlation heatmap analysis showed that metabolites involved in neurotransmission, including epinephrine and 3-methoxytyramine; cholinergic signaling and membrane lipid metabolism, including acetylcholine and phosphocholine; inflammatory lipid mediators, including prostaglandins; oxidative stress and redox balance, including oxidized glutathione; and energy and vitamin metabolism were significantly correlated with specific microbial genera (Figure 9d).

Together, these correlation-based multi-omics results reveal significant associations among gut microbiota alterations, hippocampal metabolic remodeling, and neurobiological indicators. These findings support a microbiota–metabolite–hippocampus association framework related to the neurobehavioral effects of GbO-H, while causal relationships among these layers require further validation.

## 3. Discussion

Chronic stress is increasingly recognized as a systemic neuroimmune challenge rather than a purely central monoaminergic disturbance. Accumulating evidence indicates that depression- and stress-related behavioral abnormalities are closely associated with neuroendocrine dysregulation, peripheral and central inflammatory activation, oxidative imbalance, impaired neuroplasticity, metabolic remodeling, and gut microbiota dysbiosis [9,14,15]. In the present study, we investigated the effects of inhaled GbO in a CUMS mouse model using behavioral, molecular, metabolomics, and gut microbiota analyses. GbO, particularly at the higher exposure level, attenuated depression- and anxiety-like behavioral abnormalities, accompanied by coordinated changes in hippocampal neuroimmune and neurotrophic responses, metabolic profiles, and gut microbial community structure. These findings support a multi-level stress–immune–metabolism–microbiota response framework associated with the neurobehavioral effects of GbO.

The chemical profiling and in silico analyses provide compositional and hypothesis-generating context for the in vivo findings. The predicted targets and enriched functions primarily involved neuroactive ligand–receptor interactions, serotonergic signaling, cAMP signaling, synaptic localization, and neurotransmitter regulation. These functional themes are broadly consistent with the observed recovery of hippocampal 5-HT and GABA levels, neurotrophic signaling, dendritic spine density, and stress-related behavior after GbO exposure. Among the prioritized targets, Nr3c1 is relevant to glucocorticoid signaling and stress adaptation, whereas Oprm1 and Oprk1, together with serotonergic, dopaminergic, and cholinergic receptors and transporters, suggest potential involvement of interconnected neuromodulatory systems. The limited number of high-confidence interactions in the PPI network further suggests that these predicted targets represent several related signaling components rather than a single densely connected molecular module. Docking provided additional structural hypotheses for selected predicted interactions, with α-cadinol–Nr3c1 showing the most favorable score among the evaluated pairs and geraniol–Oprm1 selected as a representative opioid-receptor-related interaction. However, docking scores indicate only computational compatibility and do not demonstrate biological binding or target engagement. Moreover, the compounds prioritized by BBB prediction and target prediction cannot be assumed to have entered the circulation or brain following inhalation. The computational analyses therefore serve to prioritize candidate compounds, targets, and pathways for subsequent validation, while the interpretation of the present study remains centered on the experimentally observed neuroendocrine, inflammatory, oxidative, neurotrophic, synaptic, metabolic, and microbial responses.

Behavioral abnormalities are the most direct phenotypic manifestations of chronic stress exposure. In this study, CUMS markedly decreased sucrose preference, increased immobility time in the TST and FST, and reduced open-arm exploration in the EPM and central-area exploration in the OFT, indicating the development of anhedonia, behavioral despair, and anxiety-like avoidance behavior. These multidimensional behavioral alterations are consistent with the broad stress-related phenotype induced by CUMS, which includes depression-related and anxiety-like behavioral outcomes rather than a single behavioral domain [15,16]. GbO treatment partially alleviated these behavioral abnormalities, with the GbO-H group showing the most consistent improvement. Importantly, total distance traveled in the OFT was not significantly altered among groups, suggesting that the observed behavioral effects were not simply attributable to changes in general locomotor activity. These findings indicate that inhaled GbO attenuated multiple chronic stress-induced behavioral outcomes rather than producing nonspecific motor stimulation. Comparable antidepressant-like effects have been reported for other inhaled aromatic volatile oils in chronic stress models. Bergamot essential oil inhalation has been reported to ameliorate CUMS-induced depression-like behavior in rats in association with preserved hippocampal neuronal plasticity, whereas agarwood essential oil inhalation reduced depression-like behaviors in CUMS mice together with modulation of the Glu/GABA neurotransmitter system [17,18]. More recently, inhalation aromatherapy was shown to alleviate CUMS-induced depression-like behavior and inflammatory responses involving the peripheral olfactory system [10]. Compared with these reports, the present study further links behavioral improvement following volatile-oil inhalation with hippocampal neuroimmune and neurotrophic changes, metabolic remodeling, and gut microbiota alterations.

At the peripheral and neuroendocrine levels, CUMS increased serum CORT and pro-inflammatory cytokines, including TNF-α, IL-6, and IL-1β. These changes are consistent with the concept that chronic stress disrupts hypothalamic–pituitary–adrenal axis activity and promotes peripheral inflammatory activation [1,19]. GbO treatment reduced serum CORT and cytokine levels, suggesting that its behavioral effects were accompanied by attenuation of systemic stress and inflammatory responses. The simultaneous reduction in peripheral inflammatory mediators and hippocampal inflammatory cytokines is particularly relevant for a brain–immune interaction framework, because peripheral immune activation can influence central inflammatory tone and neuronal function through multiple communication routes, including humoral inflammatory mediators, neuroendocrine signals, and gut–brain interactions [20,21,22]. Although the present study did not directly examine blood–brain barrier integrity or immune cell trafficking, the parallel changes in serum and hippocampal inflammatory markers support a close association between peripheral inflammatory tone and hippocampal neuroimmune status under CUMS and GbO treatment conditions.

Hippocampal glial activation represents a key neuroimmune feature of chronic stress-related behavioral dysfunction. Chronic stress can activate microglia and astrocytes, leading to excessive cytokine production, amplification of inflammatory signaling, and disruption of neuronal and synaptic homeostasis [5,23]. In the present study, CUMS increased Iba-1 and GFAP fluorescence intensities in the hippocampus, accompanied by elevated expression of inflammatory and glial activation-related genes, including Il1b, Il6, Tnf, Aif1, and Gfap. These findings indicate that chronic stress enhanced microglial activation and astrocyte reactivity in the hippocampus. GbO treatment reduced Iba-1 and GFAP signals and downregulated inflammatory gene expression, particularly in the GbO-H group. Western blot analysis further showed that CUMS increased the p-NF-κB/NF-κB ratio and decreased Nrf2 and HO-1 protein levels, whereas GbO attenuated NF-κB phosphorylation and increased Nrf2/HO-1 expression. NF-κB is a central inflammatory signaling node involved in cytokine transcription, whereas Nrf2/HO-1 is a major antioxidant defense pathway that can counterbalance oxidative and inflammatory stress [24,25,26]. These pathways should not be interpreted as a simple linear cascade in this study; rather, they reflect functionally coupled inflammatory–oxidative signaling states. Together with the biochemical data showing decreased GSH, SOD, and CAT and increased MDA after CUMS, the molecular results suggest that GbO helped rebalance hippocampal inflammatory and oxidative responses associated with chronic stress.

In addition to neuroimmune activation, CUMS induced hippocampal morphological and synaptic alterations. The hippocampus is highly sensitive to chronic stress, and stress-related inflammatory and oxidative disturbances can impair neuronal homeostasis, dendritic architecture, and synaptic plasticity [27,28]. H&E and Nissl staining showed observable hippocampal morphological changes, including less organized neuronal arrangement and attenuated Nissl staining patterns in CA1, CA3, and DG regions. These findings should be interpreted as supportive histological evidence of stress-associated hippocampal alterations rather than definitive proof of severe neuronal damage. Golgi staining provided more direct structural evidence by showing that CUMS significantly reduced hippocampal dendritic spine density, whereas Flu and GbO treatment partially restored spine density. Because dendritic spines are major postsynaptic structures involved in excitatory synaptic transmission, their reduction is closely linked to impaired hippocampal plasticity and stress-related behavioral abnormalities [29,30]. The partial recovery of spine density after GbO treatment suggests that improved synaptic structural plasticity may be an important component of the observed neurobehavioral effects.

The neurotrophic and synaptic marker data further support this interpretation. The BDNF/TrkB/CREB signaling axis plays a central role in neuronal survival, synaptic maintenance, and stress-related neuroplasticity [31,32,33]. In the present study, the coordinated recovery of BDNF/TrkB/CREB-related signaling and PSD95-associated synaptic integrity after GbO treatment was consistent with the partial restoration of dendritic spine density, linking molecular and structural indices of hippocampal plasticity. From a neuroimmune perspective, this relationship is particularly relevant because inflammatory and oxidative stress can suppress neurotrophic signaling and destabilize synaptic architecture [34,35]. Thus, improved neuroplasticity may represent a downstream or parallel correlate of the rebalanced hippocampal inflammatory–oxidative state observed after GbO treatment. However, because pathway inhibition or genetic manipulation was not performed, these findings should be interpreted as pathway-associated evidence rather than proof that BDNF/TrkB/CREB signaling is required for the effects of GbO.

Hippocampal metabolomics provided an additional systems-level perspective on the biological changes associated with CUMS and GbO-H treatment. Metabolic dysregulation in depression and chronic stress involves interconnected disturbances in neurotransmitter metabolism, lipid remodeling, redox balance, mitochondrial energy metabolism, and inflammatory lipid signaling [2,36,37,38,39]. In the present study, GbO-H partially shifted the stress-altered hippocampal metabolic profile toward the Control pattern, with the affected metabolic features converging primarily on neurotransmitter regulation, membrane and lipid metabolism, and energy/redox homeostasis. These functional domains are closely related to the neuroimmune and synaptic phenotypes observed in parallel. For example, arachidonic acid-derived inflammatory lipid mediators can participate in cytokine signaling and glial responses [40], whereas glycerophospholipid remodeling may influence synaptic membrane organization, receptor function, and neurotransmission [41,42]. Thus, the metabolomic findings provide biochemical context for the inflammatory–oxidative and neuroplasticity-related changes observed in the hippocampus rather than representing an isolated metabolic response.

Gut microbiota analysis further extended the stress-associated response framework beyond the hippocampus. Gut microbial dysbiosis has been implicated in depression and stress-related disorders through its effects on immune regulation, microbial metabolite production, neurotransmitter-related metabolism, and gut–brain communication [20,21,43,44]. In the present study, CUMS was associated with reduced microbial diversity and marked alterations in community composition, whereas GbO-H partially shifted the microbial profile toward the Control pattern. Among these changes, the CUMS-associated increase in Proteobacteria is consistent with its frequent association with microbial dysbiosis and an unstable or inflammatory gut environment [45], while the reductions in Actinobacteriota and Firmicutes may reflect disruption of microbial groups broadly involved in host metabolic regulation and gut homeostasis [46,47]. At the genus level, the partial recovery of taxa such as Lactobacillus after GbO-H treatment may also be relevant to restoration of microbial ecological balance. Nevertheless, 16S rRNA sequencing provides taxonomic rather than direct functional information; therefore, these compositional changes should be interpreted as evidence of microbial ecological remodeling rather than proof of specific microbial functions.

Microbial correlation network analysis showed that CUMS reduced network size and connectivity, whereas GbO-H partially restored network complexity. Because these networks were derived from Spearman correlations, they represent association patterns rather than direct ecological interactions. Thus, the network findings complement the taxonomic analyses by suggesting that GbO-H was associated with partial reorganization of stress-disrupted microbial community structure [48,49].

Correlation-based multi-omics analysis linked microbial shifts with hippocampal metabolism and neurobiological indices. Several genera correlated with inflammatory cytokines, oxidative indices, GABA, 5-HT, BDNF, and CORT, and Procrustes analysis showed significant concordance between microbiota and hippocampal metabolomic profiles. Microbiota–metabolite networks further connected specific taxa with metabolites involved in neurotransmission, lipid signaling, inflammatory mediators, redox balance, and energy/vitamin metabolism [50,51]. These data support a microbiota–metabolite–hippocampus association framework, but they do not establish causal directionality; microbiota-targeted or metabolite-intervention studies will be required to test causality.

The inhalation route of GbO administration deserves specific consideration. Essential oils are volatile and lipophilic mixtures, and inhalation exposure may influence neurobehavioral outcomes through olfactory pathways, respiratory absorption, systemic circulation, or stress-modulatory sensory effects [52,53]. In the present study, GbO inhalation was associated with reduced behavioral abnormalities and coordinated neuroimmune, neurotrophic, metabolic, and microbial changes. However, the 10 and 30 μL treatments represent nominal chamber-loading volumes rather than measured airborne concentrations or actual inhaled doses. The airborne composition may differ from the composition of the original oil because individual constituents vary in volatility and chamber behavior. Consequently, the present data do not establish which constituents were inhaled, their respective exposure levels, or whether the compounds prioritized by the computational analysis reached the circulation or brain. The relative contributions of olfactory stimulation and systemic pharmacological exposure also remain unresolved. Future studies combining chamber-air analysis with targeted measurement of representative constituents in plasma and brain tissue will be important for relating the nominal exposure conditions to internal exposure and biological activity.

Several limitations should be acknowledged. The multi-omics relationships are correlational and do not establish causal links among gut microbiota, hippocampal metabolism, neuroinflammation, and behavior. Likewise, although NF-κB-, Nrf2/HO-1-, and BDNF/TrkB/CREB-related changes were observed, pathway inhibition or cell-specific manipulation was not performed. The network-pharmacology and molecular-docking analyses were used for exploratory prioritization and require experimental target-engagement and pharmacokinetic validation. In particular, the absence of measured chamber-air concentrations and internal exposure data prevents individual GbO constituents from being directly linked to the observed effects. In addition, only male mice were included in the present study. Given the well-recognized sex differences in the prevalence and neurobiological features of depression, as well as in behavioral and neuroimmune responses to chronic stress, the extent to which the present findings can be generalized to females remains uncertain. Finally, H&E and Nissl staining provide supportive rather than quantitative evidence of hippocampal structural alterations. Future studies should therefore combine controlled exposure characterization and component-level pharmacokinetics with pathway- and microbiota-targeted interventions, quantitative histological assessment, and evaluation in both sexes.

## 4. Materials and Methods

### 4.1. Essential Oil Extraction and GC–MS Analysis

#### 4.1.1. Essential Oil Extraction

Fresh *Ginkgo biloba* leaves were collected in Jilin City, Jilin Province, China, in August 2024. The botanical identity was authenticated by Jinghua Li, and a voucher specimen (No. JM-202402-B) was deposited in the Traditional Chinese Medicine Herbarium of Jilin Medical University, Jilin, China. The leaves were cleaned to remove surface debris, air-dried in the shade at room temperature, and cut into small pieces. *Ginkgo biloba* leaf essential oil (GbO) was extracted by hydrodistillation using a Clevenger-type apparatus. Briefly, 100 g of dried leaf material was immersed in 1000 mL of distilled water and distilled for 3 h under gentle boiling. The obtained essential oil layer was separated, dried over anhydrous sodium sulfate, and stored in amber vials at 4 °C until further analysis. The essential-oil yields of the three independent extractions ranged from 0.14% to 0.18% (*w*/*w*). The extracted GbO was used for subsequent GC–MS analysis and animal experiments.

#### 4.1.2. HS-SPME–GC–MS Analysis

Three independently extracted batches of GbO were analyzed, of which Batch 1 was the batch used in the animal inhalation experiment. For each batch, 2 mL of undiluted GbO was transferred into a 20 mL headspace vial, and 2 μL of 2,4,6-trimethylpyridine solution (10 μg/mL) was added as an internal standard. After equilibration at 50 °C for 15 min, volatile compounds were extracted from the headspace for 30 min using a 50/30 μm DVB/CAR/PDMS fiber and thermally desorbed in the GC inlet at 250 °C for 5 min under splitless conditions.

Analysis was performed using a Thermo Fisher Scientific TSQ 9000 GC–MS system equipped with an HP-5MS capillary column (30 m × 0.25 mm i.d. × 0.25 μm film thickness; 5%-phenyl-methylpolysiloxane). Helium (≥99.999%) was used at a constant flow rate of 1.0 mL/min. The oven was maintained at 40 °C for 3 min, heated to 150 °C at 5 °C/min and then to 250 °C at 10 °C/min, and held at 250 °C for 5 min. Mass spectra were acquired in electron-ionization mode at 70 eV over m/z 35–550. The ion-source, transfer-line, and quadrupole temperatures were 230, 280, and 150 °C, respectively.

Mass spectra were searched against the NIST 21 and Wiley 8n libraries, with a match factor >85% used for initial spectral screening. Experimental linear retention indices (*RIexp*) were determined using a C7–C30 n-alkane mixture (Sigma–Aldrich, St. Louis, MO, USA) analyzed under identical chromatographic conditions and calculated according to the van den Dool and Kratz equation, *RI_exp_* = 100*n* + 100(*t_x_* − *t_n_*)/(*t_n_*_+1_ − *t_n_*), where *t_x_* is the analyte retention time and *t_n_* and *t_n_*_+1_ are the retention times of the bracketing n-alkanes. All analytes fell within the C7–C30 interpolation range, and no extrapolation was required. Compound assignments were evaluated using mass-spectral similarity, agreement between *RI_exp_* and literature RI values reported for comparable 5%-phenyl stationary phases, and reproducibility across the three independent extraction batches. In the absence of authentic-standard confirmation, the assignments were considered tentative. Adjacent chromatographic peaks were evaluated individually and were not combined solely on the basis of similar library assignments.

Constituent levels were estimated from analyte-to-internal-standard peak-area ratios using 2,4,6-trimethylpyridine as the internal standard. Because compound-specific calibration curves and response factors were unavailable, the results were treated as internal-standard-based semiquantitative estimates rather than absolute concentrations. Results across the three independent extraction batches are reported as the mean, standard deviation, relative standard deviation, and detection frequency.

### 4.2. Exploratory Network Pharmacology Analysis

GbO constituents reproducibly detected across the three independent extraction batches and supported by mass-spectral and retention-index evaluation were included in the exploratory network pharmacology analysis. Their canonical SMILES were obtained from PubChem and evaluated using the BOILED-Egg model in SwissADME (https://www.swissadme.ch/). Compounds predicted to be BBB-permeable were retained for subsequent computational screening. BBB permeability prediction was used solely for in silico prioritization and was not considered evidence of actual brain exposure.

Potential targets were predicted using SwissTargetPrediction (https://www.swisstargetprediction.ch/) with *Mus musculus* selected as the species. Predictions with probability values greater than zero were retained and standardized using mouse gene symbols. Duplicate compound–target records were removed without imposing an arbitrary limit on the number of targets retained for each compound.

Depressive disorder-associated genes were obtained from the Mouse Genome Informatics/Alliance of Genome Resources disease-association dataset using the hierarchical Disease Ontology term “depressive disorder” (DOID:1596). The retrieved records were standardized to mouse gene symbols and deduplicated. Because these records represented human disease associations mapped to mouse orthologs, they were used as a comparative disease-gene reference set rather than as direct evidence from experimental mouse models. Overlap between the disease-associated gene set and the predicted compound targets was determined, and the compound–target network was constructed and visualized using Cytoscape version 3.10.4.

Protein–protein interaction analysis of the shared genes was performed using STRING version 12.0, with *Mus musculus* selected as the organism and a minimum required interaction score of 0.700. Gene Ontology enrichment analysis, covering biological process, cellular component, and molecular function categories, and Kyoto Encyclopedia of Genes and Genomes pathway enrichment analysis were performed using STRING. Terms with false-discovery-rate-adjusted *p*-values below 0.05 were considered significantly enriched.

### 4.3. Molecular Docking Analysis

The three-dimensional protein structures used for NR3C1 and OPRM1 were obtained from the Protein Data Bank (PDB), and ligand structures were retrieved from PubChem. Before docking, co-crystallized ligands and water molecules were removed from the protein structures, polar hydrogen atoms and charges were added, and the receptor and ligand structures were converted to PDBQT format. Ligand structures were subjected to energy minimization before docking. Molecular docking was performed using AutoDock Vina version 1.1.2. The docking grid was centered on the co-crystallized ligand-binding site when available or on the previously reported functional binding pocket. For each ligand–target pair, the pose with the lowest predicted binding energy was selected for visualization and interaction analysis. Docking scores are reported in kcal/mol, with more negative values indicating more favorable predicted binding. The docking results were used to assess potential structural compatibility and were not considered evidence of in vivo target engagement.

### 4.4. Animals and Experimental Design

SPF male C57BL/6J mice (8 weeks old, 18–22 g) were obtained from Changchun Yisi Co., Ltd. (Changchun, China). All animal experiments in this study were conducted in strict accordance with the Regulations for the Administration of Laboratory Animals and relevant guidelines for animal welfare and ethics, and were reviewed and approved by the Animal Ethics Committee of Jilin Medical University (Approval No. 2024-GKJJ-019; 11 March 2024). After acclimation, baseline sucrose preference was assessed before stress exposure to confirm comparability among animals. Mice were then randomly allocated into five experimental groups (n = 12 per group): Control, CUMS, CUMS + fluoxetine (Flu), CUMS + GbO low exposure (GbO-L), and CUMS + GbO high exposure (GbO-H).

Depression-like behaviors were induced using a 6-week chronic unpredictable mild stress (CUMS) paradigm. During the modeling period, mice in the CUMS groups were exposed to one stressor per day in a variable sequence, and the same stressor was not administered on consecutive days to maintain unpredictability. Stressors included cage tilting, wet bedding, transient food or water deprivation, disruption of the light/dark cycle, restraint, cage shaking, cold-water swimming, and tail pinch. Control mice were handled routinely without stress exposure. Sucrose preference was monitored during the CUMS procedure to evaluate modeling progression.

Fluoxetine was dissolved in distilled water and administered by oral gavage at 10 mg/kg/day as the pharmacological reference treatment. Mice in the other groups received an equivalent volume of distilled water by oral gavage. GbO was administered by passive inhalation once daily using a closed acrylic exposure chamber. Treatments were initiated on day 28 after the beginning of CUMS exposure and continued until day 42, while stress exposure was maintained throughout the treatment period.

For GbO inhalation, 10 μL (GbO-L) or 30 μL (GbO-H) of freshly prepared essential oil was applied to a cotton pad placed in a perforated holder inside the exposure chamber. The cotton pad was separated from the animals to prevent direct oral or dermal contact. Mice were exposed to GbO vapor for 30 min per day within a fixed daily time window. Control, CUMS, and Flu-treated mice underwent the same chamber procedure using a blank cotton pad.

The exposure chamber measured 32 × 24 × 32 cm. For each treatment group, the 12 mice were randomly distributed among four identical chambers, with three mice placed in each chamber, and all four chambers were operated simultaneously during each exposure session. Chamber assignments and three-mouse combinations were re-randomized across exposure days rather than maintained as fixed exposure cohorts. The chambers remained closed during the 30 min exposure period without forced airflow and were opened immediately afterward for ventilation. Cotton pads were freshly prepared before each session and discarded after exposure. Different treatment groups underwent the chamber procedure in separate sessions, and the chambers were thoroughly cleaned and ventilated between groups to minimize cross-exposure. The GbO-L and GbO-H conditions therefore represent nominal chamber-loading volumes of 10 and 30 μL, respectively, rather than measured airborne concentrations or actual inhaled doses.

After completion of behavioral testing, mice were euthanized for sample collection. Brains were rapidly removed on ice; part of the tissue was snap-frozen in liquid nitrogen and stored at −80 °C for subsequent analyses, whereas the remaining tissue was fixed or preserved for histological and morphological examination. Blood was collected for serum preparation, and serum aliquots and fresh fecal pellets were stored at −80 °C until subsequent analyses.

### 4.5. Behavioral Assays

Behavioral tests were performed after completion of the CUMS procedure in a dedicated behavioral room under standardized conditions. Mice were habituated to the testing environment for at least 30 min before each test. All assessments were conducted by investigators blinded to group allocation, and the tests were performed in a fixed order to minimize potential carryover effects.

#### 4.5.1. Open Field Test (OFT)

Locomotor activity and exploratory behavior were evaluated using an open field apparatus. Each mouse was placed in the center of the arena and allowed to explore freely for 5 min. Total distance traveled and time spent in the central zone were recorded using an automated video-tracking system.

#### 4.5.2. Elevated Plus Maze (EPM)

Anxiety-like behavior was assessed using an elevated plus maze consisting of two open arms and two closed arms elevated above the floor. Each mouse was placed in the center facing an open arm and allowed to explore for 5 min. Time spent in the open arms and the number of open-arm entries were recorded.

#### 4.5.3. Sucrose Preference Test (SPT)

Anhedonia-like behavior was assessed by the sucrose preference test. Mice were habituated to sucrose solution before testing. During the test, each mouse was provided with two pre-weighed bottles containing sucrose solution and water for 24 h. Bottle positions were switched halfway through the test to avoid side preference. Sucrose preference was calculated as: sucrose preference (%) = sucrose intake/(sucrose intake + water intake) × 100%.

#### 4.5.4. Tail Suspension Test (TST)

Depression-like behavior was evaluated using the tail suspension test. Mice were suspended by the tail with adhesive tape attached to a horizontal bar for 6 min. Immobility time during the final 4 min was quantified.

#### 4.5.5. Forced Swim Test (FST)

The forced swim test was performed to assess behavioral despair. Mice were individually placed in a transparent cylinder filled with water at 23–25 °C for 6 min. Immobility time during the final 4 min was recorded. After testing, mice were dried and returned to their home cages under warming conditions.

### 4.6. Histological Staining, Golgi Staining, and Immunofluorescence

#### 4.6.1. Hematoxylin and Eosin (H&E) Staining

Paraffin-embedded sections were deparaffinized in xylene, rehydrated through a graded ethanol series, stained with hematoxylin and eosin, dehydrated, cleared, and mounted. Histological images were acquired using a light microscope under identical settings.

#### 4.6.2. Nissl Staining

Paraffin sections were deparaffinized, rehydrated, and stained with Nissl staining solution to evaluate neuronal morphology and Nissl bodies. After differentiation, sections were dehydrated, cleared, and mounted. Images were captured using a light microscope for subsequent analysis.

#### 4.6.3. Golgi Staining and Dendritic Spine Analysis

Golgi staining was performed using a commercial Golgi staining kit based on the Golgi–Cox method according to the manufacturer’s instructions. Briefly, fresh brain tissues were immersed in impregnation solution for the recommended duration and then transferred to post-impregnation solution. Tissues were sectioned, stained, dehydrated, cleared, and mounted. Representative dendritic morphology and dendritic spines were observed under a microscope. Dendritic spine density was quantified using ImageJ software (version 1.54k; National Institutes of Health, Bethesda, MD, USA) by investigators blinded to group allocation.

#### 4.6.4. Immunofluorescence Staining

Immunofluorescence staining was performed to assess microglial activation (Iba1), astrocyte reactivity (GFAP), neuronal integrity (NeuN), and neurotrophic signaling (BDNF). After antigen retrieval, brain sections were blocked with normal serum and incubated overnight at 4 °C with primary antibodies. After washing, sections were incubated with fluorescence-conjugated secondary antibodies at room temperature in the dark. Nuclei were counterstained with DAPI, and slides were mounted using anti-fade mounting medium. Fluorescence images were acquired using a fluorescence microscope under identical acquisition parameters.

### 4.7. Western Blot

Total protein was extracted using RIPA lysis buffer supplemented with protease and phosphatase inhibitors, and protein concentrations were determined by BCA assay. Equal amounts of protein were mixed with loading buffer, denatured, separated by SDS–PAGE, and transferred onto PVDF membranes. Membranes were blocked with 5% non-fat milk in TBST at room temperature and incubated overnight at 4 °C with primary antibodies against p-NF-κB, NF-κB, HO-1, Nrf2, BDNF, p-TrkB, TrkB, p-CREB, CREB, PSD95, and GAPDH. After TBST washing, membranes were incubated with HRP-conjugated secondary antibodies for 1 h at room temperature. Protein bands were visualized using enhanced chemiluminescence (ECL) reagent and imaged with a chemiluminescence imaging system. Band intensities were quantified using ImageJ. Total protein targets were normalized to GAPDH, and phosphorylated proteins were expressed as ratios to their corresponding total proteins.

### 4.8. Serum and Hippocampal Biochemical Assays

Serum corticosterone (CORT), as well as IL-1β, TNF-α, and IL-6 levels in serum and hippocampal homogenates, were quantified using commercial ELISA kits according to the manufacturers’ instructions. Hippocampal levels of γ-aminobutyric acid (GABA), 5-hydroxytryptamine (5-HT), BDNF, glutathione (GSH), superoxide dismutase (SOD), catalase (CAT), and malondialdehyde (MDA) were similarly determined using the corresponding commercial ELISA kits. Absorbance was measured using a microplate reader, and analyte concentrations were calculated from the standard curve established for each assay, with the appropriate sample dilution factors applied. Detailed information on the assay kits, including assay types, manufacturers, catalog numbers, and sample types, is provided in Table S6.

### 4.9. Untargeted LC–MS-Based Metabolomics

Frozen hippocampal samples from the Control, CUMS, and GbO-H groups were subjected to untargeted metabolomic profiling. Approximately 50 ± 5 mg of tissue was extracted with 400 μL of methanol/water (4:1, *v*/*v*) containing internal standards. Samples were homogenized under low-temperature conditions, ultrasonically extracted, incubated at −20 °C for 30 min, and centrifuged at 13,000× *g* for 15 min at 4 °C. The supernatants were collected for LC–MS analysis. A pooled quality control (QC) sample was prepared by mixing equal aliquots of all sample extracts and was injected periodically to monitor analytical stability.

Metabolomic analysis was performed using a Vanquish Horizon UHPLC system coupled to a Q Exactive HF-X mass spectrometer (Thermo Fisher Scientific, Waltham, MA, USA) with an ACQUITY UPLC HSS T3 column (100 mm × 2.1 mm, 1.8 μm; Waters Corporation, Milford, MA, USA). Data were acquired in both positive and negative electrospray ionization modes. Raw data were processed using Progenesis QI v3.0 (Waters Corporation, Milford, MA, USA) for peak detection, alignment, normalization, and metabolite annotation. After Pareto scaling, orthogonal partial least squares discriminant analysis (OPLS-DA) was performed to evaluate group separation. Differential metabolites were screened using variable importance in projection (VIP > 1.0) combined with univariate analysis (*p* < 0.05), followed by KEGG-based pathway enrichment analysis.

### 4.10. 16S rRNA Gene Sequencing and Gut Microbiota

Fresh fecal samples were collected under sterile conditions and stored at −80 °C until analysis. Microbial genomic DNA was extracted, and the V3–V4 region of the bacterial 16S rRNA gene was amplified using primers 338F (5′-ACTCCTACGGGAGGCAGCAG-3′) and 806R (5′-GGACTACHVGGGTWTCTAAT-3′). PCR products were purified, quantified, pooled at equimolar concentrations, and sequenced on an Illumina NextSeq 2000 platform. The raw sequencing data have been deposited in the NCBI Sequence Read Archive under BioProject accession number PRJNA1416588.

After quality filtering and read assembly, sequences were clustered into operational taxonomic units (OTUs) at 97% sequence similarity. Taxonomic annotation was performed using the RDP Classifier against the SILVA database (Release 138) with a confidence threshold of 0.7. Alpha diversity was evaluated using richness and diversity indices, whereas beta diversity was assessed by principal coordinates analysis (PCoA) based on Bray–Curtis distances. Differentially enriched taxa were identified by LEfSe analysis, with *p* < 0.05 and an LDA score > 4 considered statistically significant. Microbial correlation networks were constructed from OTU abundance data using Spearman’s rank correlations, and correlations with |r| > 0.8 and *p* < 0.05 were retained. Networks were visualized and analyzed using Gephi (version 0.9.2).

### 4.11. Multi-Omics Correlation Analysis

Multi-omics correlation analysis was performed to explore associations among gut microbial taxa, hippocampal metabolites, and neurobiological indicators. Spearman’s rank correlation analysis was used to evaluate correlations between differentially abundant microbial genera and biochemical or neurobiological indicators, including inflammatory cytokines, oxidative stress-related indices, GABA, 5-HT, BDNF, and CORT. Procrustes analysis was conducted to assess the concordance between gut microbiota profiles and hippocampal metabolomic profiles. In addition, microbial genus–metabolite correlation networks were constructed based on Spearman correlation coefficients and visualized to identify association patterns between gut microbial alterations and representative hippocampal metabolites. Correlations with *p* < 0.05 were considered statistically significant.

### 4.12. Quantitative Real-Time PCR (qRT–PCR)

Total RNA was extracted from mouse hippocampal tissues using TRIzol reagent (Invitrogen, USA) according to the manufacturer’s instructions. RNA concentration and purity were assessed before reverse transcription. cDNA was synthesized using a commercial reverse transcription kit, and quantitative real-time PCR was performed using a SYBR Green-based master mix on a real-time PCR system. Each sample was analyzed in technical triplicates, and a no-template control was included. Genes associated with neurotrophic regulation and neuroinflammation were selected for validation, including *Il1b*, *Tnf*, *Il6*, *Aif1*, *Gfap*, *Bdnf*, *Ntrk2*, *Creb1*, and *Dlg4*. Relative mRNA expression was calculated using the 2^−ΔΔCt^ method with *Gapdh* as the internal reference. The mean relative expression of the Control group was normalized to 1, while the variability among individual biological replicates was retained for calculation of the SD. Primer sequences used in this study are listed in Table S5.

### 4.13. Statistical Analysis

Data are presented as mean ± SD. Statistical analyses were performed using GraphPad Prism version 8.0.2 (GraphPad Software, Boston, MA, USA) and R. Unless otherwise specified, differences among groups were evaluated by one-way ANOVA followed by Tukey’s multiple-comparison test for post hoc pairwise comparisons. Statistical procedures and significance criteria specific to metabolomics, gut microbiota, and correlation analyses are described in Section 4.9, Section 4.10 and Section 4.11. All statistical tests were two-sided, and *p* < 0.05 was considered statistically significant.

## 5. Conclusions

**Figure 10:**
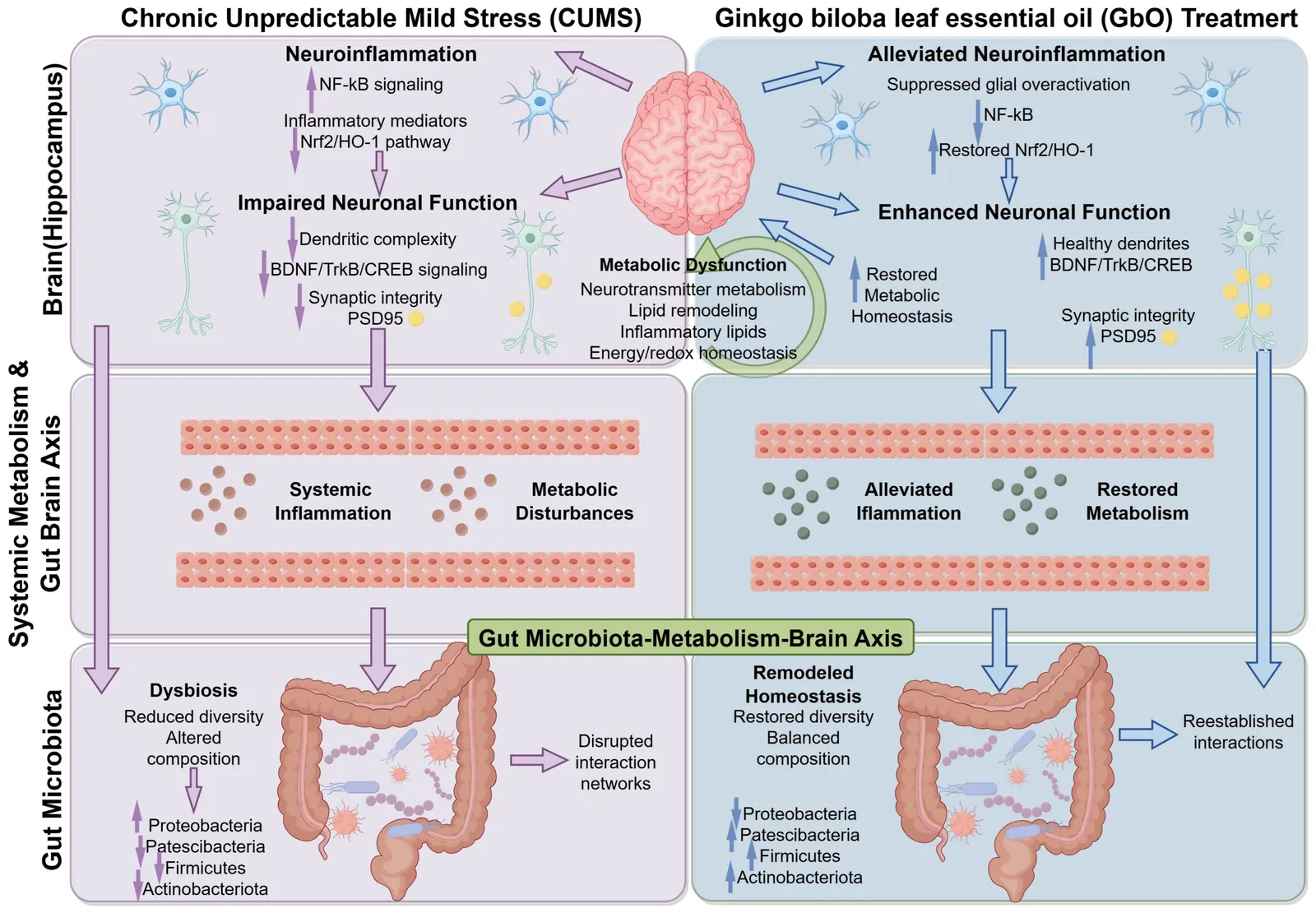
Proposed mechanistic model of the antidepressant-like effects of Ginkgo biloba leaf essential oil in CUMS mice. Chronic unpredictable mild stress induced hippocampal neuroinflammation, neuronal dysfunction, metabolic disturbances, systemic inflammatory/metabolic abnormalities, and gut microbiota dysbiosis, which were collectively associated with depression-like behaviors. GbO treatment attenuated glial overactivation and inflammatory signaling, improved Nrf2/HO-1-related antioxidant defense, restored BDNF/TrkB/CREB-related neurotrophic signaling and PSD95-associated synaptic integrity, reshaped hippocampal metabolic profiles related to neurotransmission, lipid remodeling, inflammatory lipid balance, and energy/redox homeostasis, and partially corrected gut microbiota dysbiosis by restoring microbial diversity, composition, and interaction networks. Together, these coordinated changes support the involvement of a gut microbiota–metabolism–brain axis in the antidepressant-like effects of GbO. Upward and downward arrows indicate increases and decreases, respectively, whereas outlined arrows indicate the proposed relationships among the indicated biological processes and compartments.

In conclusion, inhaled GbO attenuated CUMS-induced depression- and anxiety-like behavioral abnormalities in mice (Figure 10). These behavioral effects were accompanied by coordinated improvements in hippocampal neuroimmune, oxidative, and neurotrophic responses, together with partial remodeling of hippocampal metabolism and gut microbiota. Correlation-based multi-omics analyses further linked microbial and metabolic alterations with neurobiological indices, supporting an integrated stress–immune–metabolism–microbiota response framework. Collectively, these findings provide experimental evidence for the neurobehavioral effects of inhaled GbO under chronic stress while highlighting the need for further component-level, pharmacokinetic, and causal validation.

## Data Availability

The 16S rRNA sequencing data presented in this study are openly available in the NCBI Sequence Read Archive (SRA) under BioProject accession number PRJNA1416588. The remaining data supporting the findings of this study are available within the article and Supplementary Materials or from the corresponding author upon reasonable request.

## Supplementary Materials

The following supporting information can be downloaded at: https://www.mdpi.com/article/10.3390/plants15182833/s1, Figure S1: Total ion chromatograms (TICs) of three independent batches of Ginkgo biloba leaf essential oil analyzed by GC–MS; Table S1: Volatile constituents tentatively identified in *Ginkgo biloba* leaf essential oil by HS-SPME–GC–MS; Table S2: SwissADME properties and BBB-screening results for the 64 GbO constituents included in the exploratory computational analysis; Table S3: SwissTargetPrediction links between BBB-permeable GbO constituents and the nine depressive-disorder-associated genes; Table S4: Topological parameters of the gut microbiota co-occurrence network; Table S5: Primer sequences used for qRT-PCR analysis; Table S6: Information on commercial assay kits used in this study.

## Abbreviations

The following abbreviations are used in this manuscript:

GbO	*Ginkgo biloba* leaf essential oil
CUMS	Chronic unpredictable mild stress
GC–MS	Gas chromatography–mass spectrometry
BBB	Blood–brain barrier
MDD	Major depressive disorder
PPI	Protein–protein interaction
GO	Gene Ontology
KEGG	Kyoto Encyclopedia of Genes and Genomes
OFT	Open field test
EPM	Elevated plus maze
SPT	Sucrose preference test
TST	Tail suspension test
FST	Forced swim test
CORT	Corticosterone
GABA	γ-Aminobutyric acid
5-HT	5-Hydroxytryptamine
BDNF	Brain-derived neurotrophic factor
GSH	Glutathione
SOD	Superoxide dismutase
CAT	Catalase
MDA	Malondialdehyde
H&E	Hematoxylin and eosin
QC	Quality control
PCA	Principal component analysis
OPLS-DA	Orthogonal partial least squares discriminant analysis
VIP	Variable importance in projection
OTU	Operational taxonomic unit
PCoA	Principal coordinates analysis
LEfSe	Linear discriminant analysis effect size
qRT-PCR	Quantitative real-time polymerase chain reaction
