# Supplementary material for: Antioxidant and Anti-Inflammatory Effects of Inhaled Ginkgo biloba Leaf Essential Oil in a Chronic Stress Mouse Model: Insights from Neuroimmune, Metabolic, and Gut Microbiota Remodeling
Source: Plants (Basel). 2026 Sep 16;15(18):2833. doi: 10.3390/plants15182833 (PMC13610100; doi:10.3390/plants15182833)
Supplement: Supplementary file 1 [file plants-15-02833-s001.zip › plants-4547463-Supplementary Materials.pdf]

Supplementary Materials

Table S1. Volatile constituents tentatively identified in *Ginkgo biloba* leaf essential oil by HS-SPME–GC–MS.

No.	Compound	CAS No.	Molecular formula	RIexp (B1/B2/B3)	RIlit	Literature phase	ΔRI	Match factor (%) (B1/B2/B3)	IS-based semi-quantitative level (B1/B2/B3)	Mean ± SD	RSD (%)	Detection frequency	RI evaluation	Identification status
1	Hexanal	66-25-1	$C_6H_{12}O$	801 / 804 / 806	802	HP-5MS	1.7	94.6 / 95.7 / 93.5	14.43 / 16.06 / 12.25	14.25 ± 1.91	13.42	3/3	RI-consistent	Tentative identification (MS library match + RI agreement)
2	(Z)-3-Hexen-1-ol	928-96-1	$C_6H_{12}O$	842 / 843 / 843	851	HP-5MS	-8.3	94.7 / 94.6 / 94.7	12.31 / 20.99 / 20.36	17.89 ± 4.84	27.06	3/3	RI-consistent	Tentative identification (MS library match + RI agreement)
3	(E)-2-Hexenal	6728-26-3	$C_6H_{10}O$	852 / 847 / 848	850	HP-5MS	-1	87.8 / 88.6 / 89.0	38.70 / 38.12 / 29.93	35.58 ± 4.90	13.78	3/3	RI-consistent	Tentative identification (MS library match + RI agreement)
4	1-Hexanol	111-27-3	$C_6H_{14}O$	864 / 864 / 869	867	HP-5MS	-1.3	91.4 / 89.5 / 91.4	7.25 / 5.25 / 5.15	5.88 ± 1.18	20.14	3/3	RI-consistent	Tentative identification (MS library match + RI agreement)
5	Heptanal	111-71-7	$C_7H_{14}O$	896 / 898 / 901	899	HP-5MS	-0.7	91.2 / 88.6 / 89.7	17.74 / 12.71 / 19.42	16.62 ± 3.49	21.00	3/3	RI-consistent	Tentative identification (MS library match + RI agreement)
6	alpha-Pinene	80-56-8	$C_{10}H_{16}$	934 / 934 / 935	934	HP-5MS	0.3	91.7 / 90.7 / 91.2	10.51 / 15.93 / 11.68	12.71 ± 2.85	22.45	3/3	RI-consistent	Tentative identification (MS library match + RI agreement)
7	Camphene	79-92-5	$C_{10}H_{16}$	949 / 949 / 949	953	HP-5MS	-4	93.7 / 96.0 / 94.0	26.55 / 23.75 / 17.45	22.58 ± 4.66	20.64	3/3	RI-consistent	Tentative identification (MS library match + RI agreement)
8	Benzaldehyde	100-52-7	C_7H_6O	957 / 961 / 957	958	HP-5MS	0.3	96.8 / 96.0 / 95.6	11.70 / 15.60 / 13.72	13.67 ± 1.95	14.26	3/3	RI-consistent	Tentative identification (MS library match + RI agreement)
9	beta-Pinene	127-91-3	$C_{10}H_{16}$	970 / 976 / 972	974	HP-5MS	-1.3	94.1 / 91.1 / 92.6	45.59 / 43.10 / 42.62	43.77 ± 1.59	3.64	3/3	RI-consistent	Tentative identification (MS library match + RI agreement)
10	1-Octen-3-ol	3391-86-4	$C_8H_{16}O$	981 / 982 / 983	983	HP-5MS	-1	90.0 / 89.4 / 89.3	21.94 / 31.99 / 18.12	24.02 ± 7.16	29.83	3/3	RI-consistent	Tentative identification (MS library match + RI agreement)

11	Myrcene	123-35-3	$C_{10}H_{16}$	986 / 987 / 987	981	HP-5MS	5.7	95.6 / 94.5 / 95.0	29.46 / 31.12 / 24.84	28.47 ± 3.25	11.43	3/3	RI-consistent	Tentative identification (MS library match + RI agreement)
12	Hexanoic acid	142-62-1	$C_6H_{12}O_2$	990 / 985 / 986	999	HP-5MS	-12	90.9 / 91.3 / 91.3	21.47 / 20.14 / 19.14	20.25 ± 1.17	5.77	3/3	RI-consistent	Tentative identification (MS library match + RI agreement)
13	6-Methyl-5-hepten-2-one	110-93-0	$C_8H_{14}O$	987 / 992 / 988	988	HP-5MS	1	93.3 / 91.2 / 92.3	11.04 / 10.97 / 12.39	11.47 ± 0.80	6.98	3/3	RI-consistent	Tentative identification (MS library match + RI agreement)
14	2-Pentylfuran	3777-69-3	$C_9H_{14}O$	993 / 993 / ND	993.8	HP-5MS	-0.8	91.8 / 89.8 / ND	3.75 / 1.80 / ND	2.77 ± 1.38	—	2/3	RI-consistent	Tentative identification (MS library match + RI agreement)
15	Octanal	124-13-0	$C_8H_{16}O$	1001 / 1001 / 1002	1001	HP-5MS	0.3	91.2 / 90.0 / 91.0	3.85 / 8.10 / 6.28	6.08 ± 2.13	35.09	3/3	RI-consistent	Tentative identification (MS library match + RI agreement)
16	p-Cymene	99-87-6	$C_{10}H_{14}$	1021 / 1021 / 1021	1021	HP-5MS	0	93.1 / 91.4 / 93.0	13.55 / 10.48 / 11.34	11.79 ± 1.58	13.43	3/3	RI-consistent	Tentative identification (MS library match + RI agreement)
17	2-Ethyl-1-hexanol	104-76-7	$C_8H_{18}O$	1026 / 1022 / 1022	1026.3	HP-5MS	-3	89.2 / 90.5 / 89.0	15.15 / 14.89 / 15.28	15.11 ± 0.20	1.31	3/3	RI-consistent	Tentative identification (MS library match + RI agreement)
18	D-Limonene	5989-27-5	$C_{10}H_{16}$	1028 / 1026 / 1024	1030	HP-5MS	-4	90.6 / 90.2 / 90.4	13.15 / 13.15 / 7.60	11.30 ± 3.20	28.36	3/3	RI-consistent	Tentative identification (MS library match + RI agreement)
19	1,8-Cineole	470-82-6	$C_{10}H_{18}O$	1031 / 1027 / 1033	1031	HP-5MS	-0.7	94.7 / 93.7 / 93.4	18.56 / 20.28 / 23.45	20.76 ± 2.48	11.95	3/3	RI-consistent	Tentative identification (MS library match + RI agreement)
20	gamma-Terpinene	99-85-4	$C_{10}H_{16}$	1052 / 1053 / 1057	1060	HP-5MS	-6	92.2 / 93.0 / 91.8	8.06 / 6.96 / 6.05	7.02 ± 1.01	14.33	3/3	RI-consistent	Tentative identification (MS library match + RI agreement)
21	1-Octanol	111-87-5	$C_8H_{18}O$	1070 / 1066 / 1067	1066.9	HP-5MS	0.8	92.6 / 91.7 / 91.1	5.71 / 4.84 / 4.15	4.90 ± 0.78	15.95	3/3	RI-consistent	Tentative identification (MS library match + RI agreement)
22	Linalool	78-70-6	$C_{10}H_{18}O$	1098 / 1098 / 1095	1098	HP-5MS	-1	93.8 / 93.4 / 94.8	7.09 / 8.69 / 8.79	8.19 ± 0.95	11.65	3/3	RI-consistent	Tentative identification (MS library match + RI agreement)
23	Nonanal	124-19-6	$C_9H_{18}O$	1100 / 1106 / 1102	1103	HP-5MS	-0.3	90.0 / 90.0 / 89.6	18.70 / 16.01 / 13.06	15.92 ± 2.82	17.72	3/3	RI-consistent	Tentative identification (MS library match + RI agreement)

24	Octyl formate	112-32-3	$C_9H_{18}O_2$	1122 / 1127 / 1124	1117	DB-5	7.3	90.2 / 89.5 / 90.1	33.08 / 20.74 / 20.40	24.74 ± 7.22	29.20	3/3	RI-consistent	Tentative identification (MS library match + RI agreement)
25	Camphor	76-22-2	$C_{10}H_{16}O$	1142 / 1142 / 1143	1143	HP-5MS	-0.7	92.0 / 93.5 / 93.1	16.42 / 19.03 / 18.11	17.85 ± 1.32	7.41	3/3	RI-consistent	Tentative identification (MS library match + RI agreement)
26	Terpinen-4-ol	562-74-3	$C_{10}H_{18}O$	1173 / 1177 / ND	1175	HP-5MS	0	92.3 / 91.5 / ND	4.68 / 4.56 / ND	4.62 ± 0.08	—	2/3	RI-consistent	Tentative identification (MS library match + RI agreement)
27	alpha-Terpineol	98-55-5	$C_{10}H_{18}O$	1185 / 1181 / 1185	1186	HP-5MS	-2.3	86.6 / 86.8 / 87.9	6.77 / 10.72 / 9.86	9.12 ± 2.08	22.79	3/3	RI-consistent	Tentative identification (MS library match + RI agreement)
28	Decanal	112-31-2	$C_{10}H_{20}O$	1202 / 1208 / ND	1204	HP-5MS	1	90.3 / 89.6 / ND	2.91 / 2.58 / ND	2.75 ± 0.23	—	2/3	RI-consistent	Tentative identification (MS library match + RI agreement)
29	cis-Theaspirane	36431-72-8	$C_{13}H_{22}O$	1247 / 1242 / 1247	1302	HP-5MS	-56.7	91.1 / 89.8 / 90.5	24.77 / 22.33 / 23.17	23.42 ± 1.24	5.29	3/3	RI-inconsistent	Tentative MS assignment; RI mismatch; excluded from downstream analyses
30	Geraniol	106-24-1	$C_{10}H_{18}O$	1251 / 1248 / 1248	1250	HP-5MS	-1	94.5 / 92.7 / 92.5	13.35 / 12.35 / 7.75	11.15 ± 2.99	26.79	3/3	RI-consistent	Tentative identification (MS library match + RI agreement)
31	Bornyl acetate	76-49-3	$C_{12}H_{20}O_2$	1284 / 1284 / 1288	1285	HP-5MS	0.3	91.9 / 93.8 / 92.3	6.03 / 7.06 / 5.88	6.32 ± 0.64	10.16	3/3	RI-consistent	Tentative identification (MS library match + RI agreement)
32	Thymol	89-83-8	$C_{10}H_{14}O$	1292 / 1287 / 1293	1290	HP-5MS	0.7	91.9 / 90.7 / 90.7	11.91 / 16.15 / 13.92	13.99 ± 2.12	15.16	3/3	RI-consistent	Tentative identification (MS library match + RI agreement)
33	Tridecane	629-50-5	$C_{13}H_{28}$	1294 / ND / 1296	1300	n-alkane reference	-5	85.5 / ND / 85.5	5.42 / ND / 5.26	5.34 ± 0.11	—	2/3	RI-consistent	Tentative identification (MS library match + RI agreement)
34	Undecanal	112-44-7	$C_{11}H_{22}O$	1301 / 1304 / 1300	1301	HP-5MS	0.7	90.1 / 90.1 / 91.8	14.54 / 9.76 / 17.16	13.82 ± 3.75	27.15	3/3	RI-consistent	Tentative identification (MS library match + RI agreement)
35	(E,E)-2,4-Decadienal	25152-84-5	$C_{10}H_{16}O$	1318 / 1313 / 1318	1317	HP-5MS	-0.7	91.7 / 93.0 / 92.9	6.51 / 9.57 / 9.92	8.67 ± 1.88	21.65	3/3	RI-consistent	Tentative identification (MS library match + RI agreement)
36	alpha-Copaene	3856-25-5	$C_{15}H_{24}$	ND / 1375 / 1374	1375	HP-5MS	-0.5	ND / 98.0 / 96.3	ND / 3.30 / 3.37	3.33 ± 0.05	—	2/3	RI-consistent	Tentative identification (MS library match + RI agreement)

37	beta-Elemene	515-13-9	$C_{15}H_{24}$	1383 / 1386 / 1384	1383	HP-5MS	1.3	91.8 / 90.8 / 90.2	10.91 / 14.67 / 11.54	12.37 ± 2.01	16.28	3/3	RI-consistent	Tentative identification (MS library match + RI agreement)
38	Tetradecane	629-59-4	$C_{14}H_{30}$	1404 / 1404 / 1404	1400	n-alkane reference	4	94.0 / 91.2 / 91.5	8.58 / 7.28 / 3.82	6.56 ± 2.46	37.50	3/3	RI-consistent	Tentative identification (MS library match + RI agreement)
39	beta-Caryophyllene	87-44-5	$C_{15}H_{24}$	1417 / 1414 / 1413	1415	HP-5MS	-0.3	94.2 / 94.9 / 95.1	18.52 / 17.76 / 16.51	17.60 ± 1.01	5.77	3/3	RI-consistent	Tentative identification (MS library match + RI agreement)
40	(E)-alpha-Ionone	127-41-3	$C_{13}H_{20}O$	1422 / 1423 / 1422	1421	HP-5MS	1.3	92.3 / 93.5 / 92.5	37.64 / 36.73 / 40.48	38.28 ± 1.96	5.11	3/3	RI-consistent	Tentative identification (MS library match + RI agreement)
41	Dihydro-beta-ionone	17283-81-7	$C_{13}H_{22}O$	1428 / 1432 / 1429	1433	HP-5MS	-3.3	95.7 / 95.0 / 96.4	17.08 / 25.17 / 24.23	22.16 ± 4.42	19.97	3/3	RI-consistent	Tentative identification (MS library match + RI agreement)
42	alpha-Humulene	6753-98-6	$C_{15}H_{24}$	1452 / 1447 / 1453	1451	HP-5MS	-0.3	87.6 / 87.0 / 87.7	62.94 / 57.37 / 58.22	59.51 ± 3.00	5.04	3/3	RI-consistent	Tentative identification (MS library match + RI agreement)
43	Geranylacetone	689-67-8	$C_{13}H_{22}O$	1459 / 1458 / 1454	1454.6	HP-5MS	2.4	88.1 / 89.8 / 87.3	96.68 / 101.57 / 107.10	101.78 ± 5.21	5.12	3/3	RI-consistent	Tentative identification (MS library match + RI agreement)
44	Germacrene D	23986-74-5	$C_{15}H_{24}$	1479 / 1483 / 1479	1480	HP-5MS	0.3	90.2 / 89.8 / 89.6	5.94 / 9.27 / 8.57	7.93 ± 1.76	22.15	3/3	RI-consistent	Tentative identification (MS library match + RI agreement)
45	(E)-beta-Ionone	79-77-6	$C_{13}H_{20}O$	1492 / 1487 / 1493	1488.9	HP-5MS	1.8	86.9 / 87.8 / 86.6	33.98 / 35.38 / 29.65	33.00 ± 2.99	9.05	3/3	RI-consistent	Tentative identification (MS library match + RI agreement)
46	Pentadecane	629-62-9	$C_{15}H_{32}$	1494 / 1500 / 1500	1500	n-alkane reference	-2	92.7 / 93.1 / 91.2	3.54 / 3.95 / 3.55	3.68 ± 0.23	6.36	3/3	RI-consistent	Tentative identification (MS library match + RI agreement)
47	delta-Cadinene	483-76-1	$C_{15}H_{24}$	1522 / 1516 / 1521	1523	HP-5MS	-3.3	87.5 / 87.4 / 87.8	13.87 / 11.83 / 11.21	12.30 ± 1.39	11.31	3/3	RI-consistent	Tentative identification (MS library match + RI agreement)
48	(E)-Nerolidol	40716-66-3	$C_{15}H_{26}O$	1562 / 1567 / 1567	1566	HP-5MS	-0.7	96.4 / 96.8 / 97.3	188.09 / 178.32 / 163.13	176.51 ± 12.58	7.13	3/3	RI-consistent	Tentative identification (MS library match + RI agreement)
49	Spathulenol	6750-60-3	$C_{15}H_{24}O$	1571 / 1571 / 1571	1573	HP-5MS	-2	88.3 / 89.1 / 89.3	5.90 / 13.13 / 10.53	9.85 ± 3.66	37.17	3/3	RI-consistent	Tentative identification (MS library match + RI agreement)

50	Caryophyllene oxide	1139-30-6	$C_{15}H_{24}O$	1575 / 1581 / 1582	1580	HP-5MS	-0.7	91.3 / 91.5 / 90.0	31.87 / 32.85 / 32.97	32.56 ± 0.60	1.85	3/3	RI-consistent	Tentative identification (MS library match + RI agreement)
51	alpha-Cadinol	481-34-5	$C_{15}H_{26}O$	1657 / 1652 / 1654	1656	HP-5MS	-1.7	93.2 / 94.0 / 92.2	14.91 / 10.37 / 11.10	12.13 ± 2.44	20.10	3/3	RI-consistent	Tentative identification (MS library match + RI agreement)
52	Heptadecane	629-78-7	$C_{17}H_{36}$	1697 / 1697 / 1702	1700	n-alkane reference	-1.3	88.3 / 87.9 / 88.4	12.03 / 16.13 / 16.53	14.90 ± 2.49	16.72	3/3	RI-consistent	Tentative identification (MS library match + RI agreement)
53	Hexahydrofarnesyl acetone	502-69-2	$C_{18}H_{36}O$	1851 / 1847 / 1847	1846.7	HP-5MS	1.6	87.1 / 87.0 / 87.8	380.07 / 362.90 / 376.07	373.01 ± 8.98	2.41	3/3	RI-consistent	Tentative identification (MS library match + RI agreement)
54	Nonadecane	629-92-5	$C_{19}H_{40}$	1903 / 1903 / 1904	1900	n-alkane reference	3.3	91.4 / 92.7 / 91.5	12.52 / 9.45 / 13.77	11.91 ± 2.22	18.66	3/3	RI-consistent	Tentative identification (MS library match + RI agreement)
55	Farnesyl acetone	1117-52-8	$C_{18}H_{30}O$	1921 / 1922 / 1923	1919.8	HP-5MS	2.2	92.2 / 91.8 / 91.5	29.91 / 29.64 / 22.73	27.43 ± 4.07	14.84	3/3	RI-consistent	Tentative identification (MS library match + RI agreement)
56	Isophytol	505-32-8	$C_{20}H_{40}O$	1941 / 1943 / 1948	1948.6	HP-5MS	-4.6	93.5 / 93.6 / 94.3	10.51 / 11.43 / 13.22	11.72 ± 1.38	11.76	3/3	RI-consistent	Tentative identification (MS library match + RI agreement)
57	Palmitic acid	57-10-3	$C_{16}H_{32}O_2$	1968 / 1968 / 1963	1969	HP-5MS	-2.7	93.2 / 92.9 / 94.5	96.03 / 85.88 / 65.27	82.39 ± 15.67	19.02	3/3	RI-consistent	Tentative identification (MS library match + RI agreement)
58	Ethyl palmitate	628-97-7	$C_{18}H_{36}O_2$	1989 / 1995 / 1989	1993	HP-5MS	-2	93.7 / 92.6 / 93.0	21.62 / 19.18 / 23.20	21.33 ± 2.03	9.49	3/3	RI-consistent	Tentative identification (MS library match + RI agreement)
59	Eicosane	112-95-8	$C_{20}H_{42}$	2006 / 2006 / 2007	2000	n-alkane reference	6.3	90.2 / 89.2 / 90.2	10.37 / 10.65 / 11.12	10.71 ± 0.38	3.54	3/3	RI-consistent	Tentative identification (MS library match + RI agreement)
60	Methyl linolenate	301-00-8	$C_{19}H_{32}O_2$	2093 / 2093 / 2094	2100	HP-5MS	-6.7	91.4 / 89.8 / 92.9	338.16 / 380.38 / 321.64	346.73 ± 30.29	8.74	3/3	RI-consistent	Tentative identification (MS library match + RI agreement)
61	Heneicosane	629-94-7	$C_{21}H_{44}$	2103 / 2098 / 2097	2100	n-alkane reference	-0.7	91.7 / 92.4 / 92.6	31.26 / 41.25 / 40.03	37.51 ± 5.45	14.53	3/3	RI-consistent	Tentative identification (MS library match + RI agreement)
62	Phytol	150-86-7	$C_{20}H_{40}O$	2116 / 2117 / 2111	2114	HP-5MS	0.7	89.0 / 90.6 / 90.7	107.00 / 115.68 / 108.95	110.54 ± 4.55	4.12	3/3	RI-consistent	Tentative identification (MS library match + RI agreement)

63	Linoleic acid	60-33-3	$C_{18}H_{32}O_2$	2137 / 2138 / 2131	2135	HP-5MS	0.3	97.5 / 97.6 / 98.0	30.22 / 20.97 / 19.63	23.61 ± 5.77	24.43	3/3	RI-consistent	Tentative identification (MS library match + RI agreement)
64	Oleic acid	112-80-1	$C_{18}H_{34}O_2$	2145 / 2150 / 2146	2147	HP-5MS	0	92.1 / 92.4 / 92.3	56.06 / 49.45 / 52.47	52.66 ± 3.31	6.28	3/3	RI-consistent	Tentative identification (MS library match + RI agreement)
65	Geranylgeraniol	24034-73-9	$C_{20}H_{34}O$	2200 / 2191 / 2192	2201.2	VF-5MS	-6.9	88.7 / 88.7 / 88.6	13.42 / 11.02 / 9.08	11.17 ± 2.17	19.46	3/3	RI-consistent	Tentative identification (MS library match + RI agreement)
66	Docosane	629-97-0	$C_{22}H_{46}$	2205 / 2206 / 2201	2200	n-alkane reference	4	96.0 / 94.2 / 93.8	23.00 / 20.54 / 19.41	20.98 ± 1.84	8.75	3/3	RI-consistent	Tentative identification (MS library match + RI agreement)
67	Tricosane	638-67-5	$C_{23}H_{48}$	2308 / 2303 / 2310	2300	n-alkane reference	7	93.6 / 94.2 / 95.3	31.12 / 22.26 / 24.50	25.96 ± 4.61	17.75	3/3	RI-consistent	Tentative identification (MS library match + RI agreement)
68	Tetracosane	646-31-1	$C_{24}H_{50}$	2401 / 2407 / 2402	2400	n-alkane reference	3.3	93.6 / 93.5 / 91.5	51.46 / 55.79 / 50.20	52.48 ± 2.93	5.59	3/3	RI-consistent	Tentative identification (MS library match + RI agreement)
69	Pentacosane	629-99-2	$C_{25}H_{52}$	2499 / 2498 / 2501	2500	n-alkane reference	-0.7	90.6 / 92.4 / 90.5	140.78 / 137.68 / 128.59	135.68 ± 6.34	4.67	3/3	RI-consistent	Tentative identification (MS library match + RI agreement)
70	Hexacosane	630-01-3	$C_{26}H_{54}$	2595 / 2597 / 2591	2600	n-alkane reference	-5.7	89.0 / 88.9 / 89.0	40.15 / 31.90 / 35.35	35.80 ± 4.14	11.57	3/3	RI-consistent	Tentative identification (MS library match + RI agreement)

Table S2. SwissADME properties and BBB-screening results for the 64 GbO constituents included in the exploratory computational analysis.

Table S1 No.	Compound	PubChem CID	MW (g/mol)	Consensus Log P	TPSA (Å^2)	GI absorption	BBB permeant	P-gp substrate	Lipinski violations
1	Hexanal	6184	100.16	1.63	17.07	High	Yes	No	0
2	(Z)-3-Hexen-1-ol	5281167	100.16	1.28	20.23	High	Yes	No	0
3	(E)-2-Hexenal	5281168	98.14	1.44	17.07	High	Yes	No	0
4	1-Hexanol	8103	102.17	1.6	20.23	High	Yes	No	0
5	Heptanal	8130	114.19	2.06	17.07	High	Yes	No	0
6	alpha-Pinene	6654	136.23	3.64	0	Low	Yes	No	1
7	Camphene	6616	136.23	3.65	0	Low	Yes	No	1
8	Benzaldehyde	240	106.12	1.62	17.07	High	Yes	No	0
9	beta-Pinene	14896	136.23	3.63	0	Low	Yes	No	1
10	1-Octen-3-ol	18827	128.21	2.18	20.23	High	Yes	No	0
11	Myrcene	31253	136.23	3.56	0	Low	Yes	No	0
12	Hexanoic acid	8892	116.16	1.44	37.3	High	Yes	No	0
13	6-Methyl-5-hepten-2-one	9862	126.2	2.03	17.07	High	Yes	No	0
15	Octanal	454	128.21	2.45	17.07	High	Yes	No	0
16	p-Cymene	7463	134.22	3.74	0	Low	Yes	No	1
17	2-Ethyl-1-hexanol	7720	130.23	2.37	20.23	High	Yes	No	0
18	D-Limonene	440917	136.23	3.53	0	Low	Yes	No	0
19	1,8-Cineole	2758	154.25	2.7	9.23	High	Yes	No	0
20	gamma-Terpinene	7461	136.23	3.51	0	Low	Yes	No	0
21	1-Octanol	957	130.23	2.43	20.23	High	Yes	No	0
22	Linalool	6549	154.25	2.64	20.23	High	Yes	No	0

23	Nonanal	31289	142.24	2.87	17.07	High	Yes	No	0
24	Octyl formate	8176	158.24	2.71	26.3	High	Yes	No	0
25	Camphor	2537	152.23	2.44	17.07	High	Yes	No	0
27	alpha-Terpineol	17100	154.25	2.59	20.23	High	Yes	No	0
30	Geraniol	637566	154.25	2.79	20.23	High	Yes	No	0
31	Bornyl acetate	93009	196.29	3.12	26.3	High	Yes	No	0
32	Thymol	6989	150.22	2.92	20.23	High	Yes	No	0
34	Undecanal	8186	170.29	3.7	17.07	High	Yes	No	0
35	(E,E)-2,4-Decadienal	5283349	152.23	2.9	17.07	High	Yes	No	0
37	beta-Elemene	6918391	204.35	4.97	0	Low	No	No	1
38	Tetradecane	12389	198.39	6.04	0	Low	No	No	1
39	beta-Caryophyllene	5281515	204.35	4.48	0	Low	No	No	1
40	(E)-alpha-Ionone	5282108	192.3	3.43	17.07	High	Yes	No	0
41	Dihydro-beta-ionone	519382	194.31	3.41	17.07	High	Yes	No	0
42	alpha-Humulene	5281520	204.35	4.51	0	Low	No	No	1
43	Geranylacetone	1549778	194.31	3.73	17.07	High	Yes	No	0
44	Germacrene D	5317570	204.35	4.54	0	Low	No	No	1
45	(E)-beta-Ionone	638014	192.3	3.33	17.07	High	Yes	No	0
46	Pentadecane	12391	212.41	6.45	0	Low	No	No	1
47	delta-Cadinene	441005	204.35	4.32	0	Low	No	No	1
48	(E)-Nerolidol	5284507	222.37	4.32	20.23	High	Yes	No	0
49	Spathulenol	92231	220.35	3.36	20.23	High	Yes	No	0
50	Caryophyllene	1742210	220.35	3.81	12.53	High	Yes	No	0

	oxide								
51	alpha-Cadinol	10398656	222.37	3.5	20.23	High	Yes	No	0
52	Heptadecane	12398	240.47	7.26	0	Low	No	No	1
53	Hexahydrofarnesyl acetone	10408	268.48	5.98	17.07	High	No	Yes	1
54	Nonadecane	12401	268.52	8.06	0	Low	No	No	1
55	Farnesyl acetone	1711945	262.43	5.38	17.07	High	No	No	1
56	Isophytol	10453	296.53	6.5	20.23	Low	No	Yes	1
57	Palmitic acid	985	256.42	5.54	37.3	High	Yes	No	1
58	Ethyl palmitate	12366	284.48	6.22	26.3	High	No	No	1
59	Eicosane	8222	282.55	8.46	0	Low	No	No	1
60	Methyl linolenate	5319706	292.46	5.71	26.3	High	Yes	No	1
61	Heneicosane	12403	296.57	8.86	0	Low	No	No	1
62	Phytol	5280435	296.53	6.59	20.23	Low	No	Yes	1
63	Linoleic acid	5280450	280.45	5.78	37.3	High	Yes	No	1
64	Oleic acid	445639	282.46	6.07	37.3	High	No	No	1
65	Geranylgeraniol	5281365	290.48	6.11	20.23	High	No	No	1
66	Docosane	12405	310.6	9.26	0	Low	No	No	1
67	Tricosane	12534	324.63	9.66	0	Low	No	Yes	1
68	Tetracosane	12592	338.65	10.06	0	Low	No	Yes	1
69	Pentacosane	12406	352.68	10.45	0	Low	No	Yes	1
70	Hexacosane	12407	366.71	10.85	0	Low	No	Yes	1

Note: BBB, blood–brain barrier; GI, gastrointestinal; MW, molecular weight; P-gp, P-glycoprotein; TPSA, topological polar surface area. Forty-one compounds predicted as BBB permeant were retained for Mus musculus target prediction. These in silico classifications were used only for exploratory prioritization.

Table S3. SwissTargetPrediction links between BBB-permeable GbO constituents and the nine depressive-disorder-associated genes.

Compound	Mouse gene symbol	Predicted target	UniProt ID	ChEMBL ID	Target class	Probability
(Z)-3-Hexen-1-ol	Chrm2	Muscarinic acetylcholine receptor M2	Q9ERZ4	CHEMBL3197	Family A G protein-coupled receptor	0.090419
1-Hexanol	Chrm2	Muscarinic acetylcholine receptor M2	Q9ERZ4	CHEMBL3197	Family A G protein-coupled receptor	0.09002
1-Octen-3-ol	Chrm2	Muscarinic acetylcholine receptor M2	Q9ERZ4	CHEMBL3197	Family A G protein-coupled receptor	0.079125
2-Ethyl-1-hexanol	Chrm2	Muscarinic acetylcholine receptor M2	Q9ERZ4	CHEMBL3197	Family A G protein-coupled receptor	0.077372
1-Octanol	Chrm2	Muscarinic acetylcholine receptor M2	Q9ERZ4	CHEMBL3197	Family A G protein-coupled receptor	0.072982
alpha-Terpineol	Chrm2	Muscarinic acetylcholine receptor M2	Q9ERZ4	CHEMBL3197	Family A G protein-coupled receptor	0.040218
Linalool	Chrm2	Muscarinic acetylcholine receptor M2	Q9ERZ4	CHEMBL3197	Family A G protein-coupled receptor	0.039096
Nonanal	Chrm2	Muscarinic acetylcholine receptor M2	Q9ERZ4	CHEMBL3197	Family A G protein-coupled receptor	0.038166
Octanal	Chrm2	Muscarinic acetylcholine receptor M2	Q9ERZ4	CHEMBL3197	Family A G protein-coupled receptor	0.036251
Heptanal	Chrm2	Muscarinic acetylcholine receptor M2	Q9ERZ4	CHEMBL3197	Family A G protein-coupled receptor	0.034917
Hexanal	Chrm2	Muscarinic acetylcholine receptor M2	Q9ERZ4	CHEMBL3197	Family A G protein-coupled receptor	0.03316
Benzaldehyde	Chrm2	Muscarinic acetylcholine	Q9ERZ4	CHEMBL3197	Family A G protein-coupled	0.031936

		receptor M2			receptor	
6-Methyl-5-hepten-2-one	Chrm2	Muscarinic acetylcholine receptor M2	Q9ERZ4	CHEMBL3197	Family A G protein-coupled receptor	0.029479
1,8-Cineole	Chrm2	Muscarinic acetylcholine receptor M2	Q9ERZ4	CHEMBL3197	Family A G protein-coupled receptor	0.017192
Caryophyllene oxide	Chrm2	Muscarinic acetylcholine receptor M2	Q9ERZ4	CHEMBL3197	Family A G protein-coupled receptor	0.00238
Thymol	Drd2	D(2) dopamine receptor	P61168	CHEMBL3427	Family A G protein-coupled receptor	0.022962
Methyl linolenate	Drd2	D(2) dopamine receptor	P61168	CHEMBL3427	Family A G protein-coupled receptor	0.001171
Thymol	Htr1a	5-hydroxytryptamine receptor 1A	Q64264	CHEMBL3737	Family A G protein-coupled receptor	0.022735
1-Octen-3-ol	Htr3b	5-hydroxytryptamine receptor 3B	Q9JHJ5	CHEMBL3781	Ligand-gated ion channel	0.127344
1-Hexanol	Htr3b	5-hydroxytryptamine receptor 3B	Q9JHJ5	CHEMBL3781	Ligand-gated ion channel	0.118443
(Z)-3-Hexen-1-ol	Htr3b	5-hydroxytryptamine receptor 3B	Q9JHJ5	CHEMBL3781	Ligand-gated ion channel	0.116429
1-Octanol	Htr3b	5-hydroxytryptamine receptor 3B	Q9JHJ5	CHEMBL3781	Ligand-gated ion channel	0.113952
2-Ethyl-1-hexanol	Htr3b	5-hydroxytryptamine receptor 3B	Q9JHJ5	CHEMBL3781	Ligand-gated ion channel	0.112005
Linalool	Htr3b	5-hydroxytryptamine receptor 3B	Q9JHJ5	CHEMBL3781	Ligand-gated ion channel	0.070852
alpha-Terpineol	Htr3b	5-hydroxytryptamine	Q9JHJ5	CHEMBL3781	Ligand-gated ion channel	0.066156

		receptor 3B				
Geraniol	Htr3b	5-hydroxytryptamine receptor 3B	Q9JHJ5	CHEMBL3781	Ligand-gated ion channel	0.061467
Nonanal	Htr3b	5-hydroxytryptamine receptor 3B	Q9JHJ5	CHEMBL3781	Ligand-gated ion channel	0.040743
Octanal	Htr3b	5-hydroxytryptamine receptor 3B	Q9JHJ5	CHEMBL3781	Ligand-gated ion channel	0.038704
Benzaldehyde	Htr3b	5-hydroxytryptamine receptor 3B	Q9JHJ5	CHEMBL3781	Ligand-gated ion channel	0.035745
Heptanal	Htr3b	5-hydroxytryptamine receptor 3B	Q9JHJ5	CHEMBL3781	Ligand-gated ion channel	0.035081
(E,E)-2,4-Decadienal	Htr3b	5-hydroxytryptamine receptor 3B	Q9JHJ5	CHEMBL3781	Ligand-gated ion channel	0.034249
(E)-2-Hexenal	Htr3b	5-hydroxytryptamine receptor 3B	Q9JHJ5	CHEMBL3781	Ligand-gated ion channel	0.032238
Hexanal	Htr3b	5-hydroxytryptamine receptor 3B	Q9JHJ5	CHEMBL3781	Ligand-gated ion channel	0.031936
6-Methyl-5-hepten-2-one	Htr3b	5-hydroxytryptamine receptor 3B	Q9JHJ5	CHEMBL3781	Ligand-gated ion channel	0.030756
Undecanal	Htr3b	5-hydroxytryptamine receptor 3B	Q9JHJ5	CHEMBL3781	Ligand-gated ion channel	0.020905
1,8-Cineole	Htr3b	5-hydroxytryptamine receptor 3B	Q9JHJ5	CHEMBL3781	Ligand-gated ion channel	0.017868
Geranylacetone	Htr3b	5-hydroxytryptamine receptor 3B	Q9JHJ5	CHEMBL3781	Ligand-gated ion channel	0.008335
(E)-Nerolidol	Htr3b	5-hydroxytryptamine	Q9JHJ5	CHEMBL3781	Ligand-gated ion channel	0.006487

		receptor 3B				
Spathulenol	Htr3b	5-hydroxytryptamine receptor 3B	Q9JHJ5	CHEMBL3781	Ligand-gated ion channel	0.005615
alpha-Cadinol	Htr3b	5-hydroxytryptamine receptor 3B	Q9JHJ5	CHEMBL3781	Ligand-gated ion channel	0.005416
Caryophyllene oxide	Htr3b	5-hydroxytryptamine receptor 3B	Q9JHJ5	CHEMBL3781	Ligand-gated ion channel	0.002668
alpha-Terpineol	Nr3c1	Glucocorticoid receptor	P06537	CHEMBL3144	Nuclear receptor	0.208407
Linoleic acid	Nr3c1	Glucocorticoid receptor	P06537	CHEMBL3144	Nuclear receptor	0.191213
alpha-Cadinol	Nr3c1	Glucocorticoid receptor	P06537	CHEMBL3144	Nuclear receptor	0.184199
1-Octanol	Nr3c1	Glucocorticoid receptor	P06537	CHEMBL3144	Nuclear receptor	0.126268
1-Octen-3-ol	Nr3c1	Glucocorticoid receptor	P06537	CHEMBL3144	Nuclear receptor	0.110674
Geraniol	Nr3c1	Glucocorticoid receptor	P06537	CHEMBL3144	Nuclear receptor	0.091218
2-Ethyl-1-hexanol	Nr3c1	Glucocorticoid receptor	P06537	CHEMBL3144	Nuclear receptor	0.084225
1-Hexanol	Nr3c1	Glucocorticoid receptor	P06537	CHEMBL3144	Nuclear receptor	0.083108
Linalool	Nr3c1	Glucocorticoid receptor	P06537	CHEMBL3144	Nuclear receptor	0.078159
(Z)-3-Hexen-1-ol	Nr3c1	Glucocorticoid receptor	P06537	CHEMBL3144	Nuclear receptor	0.076338
Methyl linolenate	Nr3c1	Glucocorticoid receptor	P06537	CHEMBL3144	Nuclear receptor	0.064794
Octyl formate	Nr3c1	Glucocorticoid receptor	P06537	CHEMBL3144	Nuclear receptor	0.063479
(E)-Nerolidol	Nr3c1	Glucocorticoid receptor	P06537	CHEMBL3144	Nuclear receptor	0.049464
Nonanal	Nr3c1	Glucocorticoid receptor	P06537	CHEMBL3144	Nuclear receptor	0.040365
(E)-2-Hexenal	Nr3c1	Glucocorticoid receptor	P06537	CHEMBL3144	Nuclear receptor	0.039079
Octanal	Nr3c1	Glucocorticoid receptor	P06537	CHEMBL3144	Nuclear receptor	0.037284
alpha-Pinene	Nr3c1	Glucocorticoid receptor	P06537	CHEMBL3144	Nuclear receptor	0.036726
gamma-Terpinene	Nr3c1	Glucocorticoid receptor	P06537	CHEMBL3144	Nuclear receptor	0.035685
6-Methyl-5-	Nr3c1	Glucocorticoid receptor	P06537	CHEMBL3144	Nuclear receptor	0.034528

hepten-2-one						
Heptanal	Nr3c1	Glucocorticoid receptor	P06537	CHEMBL3144	Nuclear receptor	0.033631
D-Limonene	Nr3c1	Glucocorticoid receptor	P06537	CHEMBL3144	Nuclear receptor	0.033462
Hexanal	Nr3c1	Glucocorticoid receptor	P06537	CHEMBL3144	Nuclear receptor	0.032696
Benzaldehyde	Nr3c1	Glucocorticoid receptor	P06537	CHEMBL3144	Nuclear receptor	0.031786
Dihydro-beta-ionone	Nr3c1	Glucocorticoid receptor	P06537	CHEMBL3144	Nuclear receptor	0.031746
(E,E)-2,4-Decadienal	Nr3c1	Glucocorticoid receptor	P06537	CHEMBL3144	Nuclear receptor	0.031594
Bornyl acetate	Nr3c1	Glucocorticoid receptor	P06537	CHEMBL3144	Nuclear receptor	0.031326
Undecanal	Nr3c1	Glucocorticoid receptor	P06537	CHEMBL3144	Nuclear receptor	0.020703
(E)-alpha-Ionone	Nr3c1	Glucocorticoid receptor	P06537	CHEMBL3144	Nuclear receptor	0.017134
(E)-beta-Ionone	Nr3c1	Glucocorticoid receptor	P06537	CHEMBL3144	Nuclear receptor	0.017134
Camphor	Nr3c1	Glucocorticoid receptor	P06537	CHEMBL3144	Nuclear receptor	0.015311
Geranylacetone	Nr3c1	Glucocorticoid receptor	P06537	CHEMBL3144	Nuclear receptor	0.013817
Spathulenol	Nr3c1	Glucocorticoid receptor	P06537	CHEMBL3144	Nuclear receptor	0.013388
Palmitic acid	Nr3c1	Glucocorticoid receptor	P06537	CHEMBL3144	Nuclear receptor	0.004272
Caryophyllene oxide	Nr3c1	Glucocorticoid receptor	P06537	CHEMBL3144	Nuclear receptor	0.003884
Thymol	Oprk1	Kappa-type opioid receptor	P33534	CHEMBL4329	Family A G protein-coupled receptor	0.019486
(E,E)-2,4-Decadienal	Oprm1	Mu-type opioid receptor	P42866	CHEMBL2858	Family A G protein-coupled receptor	0.107446
1-Octanol	Oprm1	Mu-type opioid receptor	P42866	CHEMBL2858	Family A G protein-coupled receptor	0.104958
Geraniol	Oprm1	Mu-type opioid receptor	P42866	CHEMBL2858	Family A G protein-coupled	0.075854

					receptor	
1-Hexanol	Oprm1	Mu-type opioid receptor	P42866	CHEMBL2858	Family A G protein-coupled receptor	0.066372
1-Octen-3-ol	Oprm1	Mu-type opioid receptor	P42866	CHEMBL2858	Family A G protein-coupled receptor	0.065772
2-Ethyl-1-hexanol	Oprm1	Mu-type opioid receptor	P42866	CHEMBL2858	Family A G protein-coupled receptor	0.065772
alpha-Terpineol	Oprm1	Mu-type opioid receptor	P42866	CHEMBL2858	Family A G protein-coupled receptor	0.047525
Linalool	Oprm1	Mu-type opioid receptor	P42866	CHEMBL2858	Family A G protein-coupled receptor	0.047397
(E)-2-Hexenal	Oprm1	Mu-type opioid receptor	P42866	CHEMBL2858	Family A G protein-coupled receptor	0.039067
Nonanal	Oprm1	Mu-type opioid receptor	P42866	CHEMBL2858	Family A G protein-coupled receptor	0.038166
Octanal	Oprm1	Mu-type opioid receptor	P42866	CHEMBL2858	Family A G protein-coupled receptor	0.035412
Hexanal	Oprm1	Mu-type opioid receptor	P42866	CHEMBL2858	Family A G protein-coupled receptor	0.033473
gamma-Terpinene	Oprm1	Mu-type opioid receptor	P42866	CHEMBL2858	Family A G protein-coupled receptor	0.026366
Thymol	Oprm1	Mu-type opioid receptor	P42866	CHEMBL2858	Family A G protein-coupled receptor	0.024972
Undecanal	Oprm1	Mu-type opioid receptor	P42866	CHEMBL2858	Family A G protein-coupled receptor	0.020503
(E)-Nerolidol	Oprm1	Mu-type opioid receptor	P42866	CHEMBL2858	Family A G protein-coupled	0.013666

					receptor	
alpha-Cadinol	Oprm1	Mu-type opioid receptor	P42866	CHEMBL2858	Family A G protein-coupled receptor	0.013648
(E)-alpha-Ionone	Oprm1	Mu-type opioid receptor	P42866	CHEMBL2858	Family A G protein-coupled receptor	0.009515
(E)-beta-Ionone	Oprm1	Mu-type opioid receptor	P42866	CHEMBL2858	Family A G protein-coupled receptor	0.009515
Geranylacetone	Oprm1	Mu-type opioid receptor	P42866	CHEMBL2858	Family A G protein-coupled receptor	0.00872
Dihydro-beta-ionone	Oprm1	Mu-type opioid receptor	P42866	CHEMBL2858	Family A G protein-coupled receptor	0.005212
Spathulenol	Oprm1	Mu-type opioid receptor	P42866	CHEMBL2858	Family A G protein-coupled receptor	0.004687
Linoleic acid	Oprm1	Mu-type opioid receptor	P42866	CHEMBL2858	Family A G protein-coupled receptor	0.001656
Methyl linolenate	Oprm1	Mu-type opioid receptor	P42866	CHEMBL2858	Family A G protein-coupled receptor	0.001306
1,8-Cineole	Slc5a7	High affinity choline transporter 1	Q8BGY9	CHEMBL3013	Electrochemical transporter	0.126122
(Z)-3-Hexen-1-ol	Slc5a7	High affinity choline transporter 1	Q8BGY9	CHEMBL3013	Electrochemical transporter	0.119974
1-Hexanol	Slc5a7	High affinity choline transporter 1	Q8BGY9	CHEMBL3013	Electrochemical transporter	0.09917
Hexanal	Slc5a7	High affinity choline transporter 1	Q8BGY9	CHEMBL3013	Electrochemical transporter	0.083851
Heptanal	Slc5a7	High affinity choline	Q8BGY9	CHEMBL3013	Electrochemical transporter	0.078771

		transporter 1				
1-Octen-3-ol	Slc5a7	High affinity choline transporter 1	Q8BGY9	CHEMBL3013	Electrochemical transporter	0.074308
Octanal	Slc5a7	High affinity choline transporter 1	Q8BGY9	CHEMBL3013	Electrochemical transporter	0.073311
2-Ethyl-1-hexanol	Slc5a7	High affinity choline transporter 1	Q8BGY9	CHEMBL3013	Electrochemical transporter	0.072982
1-Octanol	Slc5a7	High affinity choline transporter 1	Q8BGY9	CHEMBL3013	Electrochemical transporter	0.069762
6-Methyl-5-hepten-2-one	Slc5a7	High affinity choline transporter 1	Q8BGY9	CHEMBL3013	Electrochemical transporter	0.063713
Nonanal	Slc5a7	High affinity choline transporter 1	Q8BGY9	CHEMBL3013	Electrochemical transporter	0.061995
Benzaldehyde	Slc5a7	High affinity choline transporter 1	Q8BGY9	CHEMBL3013	Electrochemical transporter	0.05555
(E)-2-Hexenal	Slc5a7	High affinity choline transporter 1	Q8BGY9	CHEMBL3013	Electrochemical transporter	0.044715
(E,E)-2,4-Decadienal	Slc5a7	High affinity choline transporter 1	Q8BGY9	CHEMBL3013	Electrochemical transporter	0.042354
Linalool	Slc5a7	High affinity choline transporter 1	Q8BGY9	CHEMBL3013	Electrochemical transporter	0.039096
alpha-Terpineol	Slc5a7	High affinity choline transporter 1	Q8BGY9	CHEMBL3013	Electrochemical transporter	0.037826
Hexanoic acid	Slc5a7	High affinity choline transporter 1	Q8BGY9	CHEMBL3013	Electrochemical transporter	0.037458
Camphor	Slc5a7	High affinity choline	Q8BGY9	CHEMBL3013	Electrochemical transporter	0.032662

		transporter 1				
Undecanal	Slc5a7	High affinity choline transporter 1	Q8BGY9	CHEMBL3013	Electrochemical transporter	0.019721
Bornyl acetate	Slc5a7	High affinity choline transporter 1	Q8BGY9	CHEMBL3013	Electrochemical transporter	0.013694
Dihydro-beta-ionone	Slc5a7	High affinity choline transporter 1	Q8BGY9	CHEMBL3013	Electrochemical transporter	0.00872
(E)-alpha-Ionone	Slc5a7	High affinity choline transporter 1	Q8BGY9	CHEMBL3013	Electrochemical transporter	0.008251
(E)-beta-Ionone	Slc5a7	High affinity choline transporter 1	Q8BGY9	CHEMBL3013	Electrochemical transporter	0.008251
Geranylacetone	Slc5a7	High affinity choline transporter 1	Q8BGY9	CHEMBL3013	Electrochemical transporter	0.007729
Caryophyllene oxide	Slc5a7	High affinity choline transporter 1	Q8BGY9	CHEMBL3013	Electrochemical transporter	0.00525
Palmitic acid	Slc5a7	High affinity choline transporter 1	Q8BGY9	CHEMBL3013	Electrochemical transporter	0.002437
Linoleic acid	Slc5a7	High affinity choline transporter 1	Q8BGY9	CHEMBL3013	Electrochemical transporter	0.001765
p-Cymene	Slc6a4	Sodium-dependent serotonin transporter	Q60857	CHEMBL4642	Electrochemical transporter	0.04449
Hexanoic acid	Slc6a4	Sodium-dependent serotonin transporter	Q60857	CHEMBL4642	Electrochemical transporter	0.031341
Thymol	Slc6a4	Sodium-dependent serotonin transporter	Q60857	CHEMBL4642	Electrochemical transporter	0.018312

Note: Target predictions were generated in SwissTargetPrediction with Mus musculus selected as the species. Duplicate predictions for the same

compound–gene pair were collapsed by retaining the maximum probability. The table contains all 130 links connecting 38 compounds to the nine genes shared with the MGI depressive-disorder gene set. Camphene, beta-pinene, and myrcene were among the 41 BBB-positive compounds but had no predicted link to these nine genes and therefore do not appear in this table. Prediction probabilities do not demonstrate target engagement.

Table S4. Topological parameters of the gut microbiota co-occurrence network.

Network parameter	Control	CUMS	GbO-H
Nodes (n)	493	325	500
Edges (n)	8029	4241	7691
Average degree	32.572	26.098	30.764
Average weighted degree	32.572	26.098	30.764
Network diameter	5	6	5
Network density	0.066	0.081	0.062
Modularity	0.599	0.618	0.670
Average clustering coefficient	0.631	0.740	0.677
Average path length	2.617	2.597	2.634
Positive correlations (%)	73.46	77.74	68.22
Negative correlations (%)	26.54	22.26	31.78

Table S5. Primer sequences used for qRT-PCR analysis.

Gene	Forward primer (5′−3′)	reverse primer (5′−3′)	Size
Il1b	TGGACCTTCCAGGATGAGGACA	GTTCATCTCGGAGCCTGTAGTG	148
Il6	TACCACTTCACAAGTCGGAGGC	CTGCAAGTGCATCATCGTTGTTC	116
Tnf	GGTGCCTATGTCTCAGCCTCTT	GCCATAGAACTGATGAGAGGGAG	139
Aif1	TCTGCCGTCCAAACTTGAAGCC	CTCTTCAGCTCTAGGTGGGTCT	138
Gfap	CACCTACAGGAAATTGCTGGAGG	CCACGATGTTCCTCTTGAGGTG	137
Bdnf	GGCTGACACTTTTGAGCACGTC	CTCCAAAGGCACTTGACTGCTG	133
Ntrk2	CCACGGATGTTGCTGACCAAAG	GCCAAACTTGGAATGTCTCGCC	134
Creb1	CACAGACCACTGATGGACAGCA	AGGACGCCATAACAACTCCAGG	138
Dlg4	TCAGACGGTCACGATCATCGCT	GTTGCTTCGCAGAGATGCAGTC	130
Gapdh	CATCACTGCCACCCAGAAGACTG	ATGCCAGTGAGCTTCCCGTTCAG	153

Table S6. Information on commercial assay kits used in this study.

Indicator	Sample type	Assay kit / method	Manufacturer	Catalog No.
CORT	Serum	QuickKey Pro Mouse CORT (Corticosterone) ELISA Kit	Elabscience Biotechnology Inc., Wuhan, China	E-OSEL-M0001
IL-1β	Serum and hippocampal homogenate	Mouse Interleukin-1β (IL-1β) ELISA Kit	Shanghai Enzyme-linked Biotechnology Co., Ltd., Shanghai, China	ml106733
TNF-α	Serum and hippocampal homogenate	Mouse Tumor Necrosis Factor-α (TNF-α) ELISA Kit	Shanghai Enzyme-linked Biotechnology Co., Ltd., Shanghai, China	ml002095
IL-6	Serum and hippocampal homogenate	Mouse Interleukin-6 (IL-6) ELISA Kit	Shanghai Enzyme-linked Biotechnology Co., Ltd., Shanghai, China	ml098430
GABA	Hippocampal homogenate	Mouse γ-Aminobutyric Acid (GABA) ELISA Kit	Shanghai Enzyme-linked Biotechnology Co., Ltd., Shanghai, China	ml56293-J
5-HT	Hippocampal homogenate	Mouse 5-Hydroxytryptamine (5-HT) ELISA Kit	Shanghai Enzyme-linked Biotechnology Co., Ltd., Shanghai, China	ml001891
BDNF	Hippocampal homogenate	Mouse Brain-Derived Neurotrophic Factor (BDNF) ELISA Kit	Shanghai Enzyme-linked Biotechnology Co., Ltd., Shanghai, China	ml002219
GSH	Hippocampal homogenate	Glutathione (GSH) ELISA Kit	Shanghai Enzyme-linked Biotechnology Co., Ltd., Shanghai, China	ml063305
SOD	Hippocampal homogenate	Superoxide Dismutase (SOD) ELISA Kit	Shanghai Enzyme-linked Biotechnology Co., Ltd., Shanghai, China	ml001998
CAT	Hippocampal homogenate	Catalase (CAT) ELISA Kit	Shanghai Enzyme-linked Biotechnology Co., Ltd., Shanghai, China	ml037752
MDA	Hippocampal homogenate	Malondialdehyde (MDA) ELISA Kit	Shanghai Enzyme-linked Biotechnology Co., Ltd., Shanghai, China	ml53201

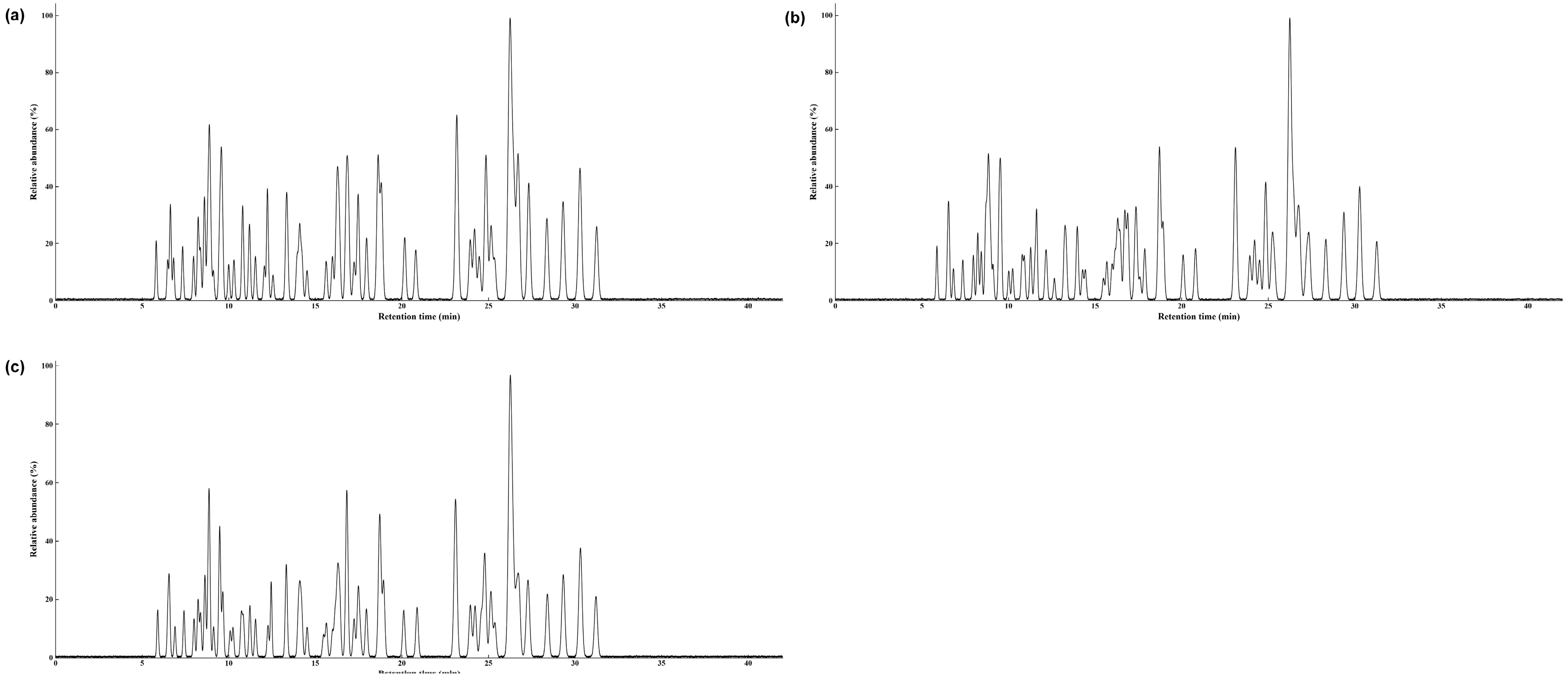

Figure S1. Total ion chromatograms (TICs) of three independent batches of *Ginkgo biloba* leaf essential oil analyzed by GC–MS. (A) The batch used for the animal inhalation experiments. (B–C) Two additional independently prepared batches.
